# The accumulation and growth of *Pseudomonas aeruginosa* on surfaces is modulated by surface mechanics via cyclic-di-GMP signaling

**DOI:** 10.1038/s41522-023-00436-x

**Published:** 2023-10-10

**Authors:** Liyun Wang, Yu-Chern Wong, Joshua M. Correira, Megan Wancura, Chris J. Geiger, Shanice S. Webster, Ahmed Touhami, Benjamin J. Butler, George A. O’Toole, Richard M. Langford, Katherine A. Brown, Berkin Dortdivanlioglu, Lauren Webb, Elizabeth Cosgriff-Hernandez, Vernita D. Gordon

**Affiliations:** 1 Department of Physics, Center for Nonlinear Dynamics, The University of Texas at Austin, Austin, TX 78712 USA; 2 Department of Mechanical Engineering, The University of Texas at Austin, Austin, TX 78712 USA; 3 Department of Chemistry, The University of Texas at Austin, Austin, TX 78712 USA; 4 Geisel School of Medicine at Dartmouth, Hanover, NH 03755 USA; 5 Department of Physics and Astronomy University of Texas Rio Grande Valley, One West University Blvd, Brownsville, TX 78520 USA; 6 Surfaces, Microstructure and Fracture Group, Cavendish Laboratory, University of Cambridge, Cambridge, CB3 0HE UK; 7 Oden Institute for Computational Engineering & Sciences, The University of Texas at Austin, Austin, TX 78712 USA; 8 Department of Civil, Architectural, and Environmental Engineering, The University of Texas at Austin, Austin, TX 78712 USA; 9 Department of Biomedical Engineering, The University of Texas at Austin, Austin, TX 78712 USA; 10 LaMontagne Center for Infectious Disease, The University of Texas at Austin, Austin, TX 78712 USA; 11 Interdisciplinary Life Sciences Graduate Program, The University of Texas at Austin, Austin, TX 78712 USA

**Keywords:** Biofilms, Applied microbiology, Pathogens

## Abstract

Attachment of bacteria onto a surface, consequent signaling, and accumulation and growth of the surface-bound bacterial population are key initial steps in the formation of pathogenic biofilms. While recent reports have hinted that surface mechanics may affect the accumulation of bacteria on that surface, the processes that underlie bacterial perception of surface mechanics and modulation of accumulation in response to surface mechanics remain largely unknown. We use thin and thick hydrogels coated on glass to create composite materials with different mechanics (higher elasticity for thin composites; lower elasticity for thick composites) but with the same surface adhesivity and chemistry. The mechanical cue stemming from surface mechanics is elucidated using experiments with the opportunistic human pathogen *Pseudomonas aeruginosa* combined with finite-element modeling. Adhesion to thin composites results in greater changes in mechanical stress and strain in the bacterial envelope than does adhesion to thick composites with identical surface chemistry. Using quantitative microscopy, we find that adhesion to thin composites also results in higher cyclic-di-GMP levels, which in turn result in lower motility and less detachment, and thus greater accumulation of bacteria on the surface than does adhesion to thick composites. Mechanics-dependent c-di-GMP production is mediated by the cell-surface-exposed protein PilY1. The biofilm lag phase, which is longer for bacterial populations on thin composites than on thick composites, is also mediated by PilY1. This study shows clear evidence that bacteria actively regulate differential accumulation on surfaces of different stiffnesses *via* perceiving varied mechanical stress and strain upon surface engagement.

## Introduction

Mechanosensing, including but not limited to responding to surface mechanics, is well-established to be an important cellular function in eukaryotes^1,2^. Much less is known about mechanosensing by prokaryotes^3–5^. Some recent studies have shown that during early biofilm formation, bacteria can sense and respond to mechanical cues, such as those arising from contacting a surface^6–12^ and varying fluid flow over surface-bound bacteria^13,14^. For the biofilm-forming pathogen *Pseudomonas aeruginosa*, previous research has shown two categories of sensing pathways. One is through cell envelope-associated proteins, the membrane-bound protein WspA, which might sense cell envelope stress upon surface attachment^12^, and the cell-surface-exposed protein PilY1, which has been proposed as a possible mechanosensor of surface adhesion^10,13^ and fluid shear^13^. PilY1 is localized at the outer membrane^9,10^ and found at the tip of type-IV pili (TFP)^15^. The second is through the extension and retraction of TFP, which power the twitching motility of *P. aeruginosa* on surfaces and are contribute to bacterial mechanosensing of surfaces^8,11^ and fluid shear^13^.

In vivo, bacteria can experience a wide range of surface mechanics, from ultrasoft (dermal fillers have elastic moduli 0.02–3 kPa and living tissues 0.2–30 kPa) to hard (orthopedic implants have elastic moduli 5–300 GPa)^16,17^. In such diverse settings, biofilm formation commonly causes chronic infection, resulting in prolonged illness and high medical costs^18–20^. Recent research has indicated connections between bacterial behavior and the physical properties of the substrates to which they are attached, as follows: Other researchers have shown that the extension and retraction of TFP of *P. aeruginosa* actively regulates virulence-related genes in a stiffness-dependent manner indicating by a *PaQa* reporter^21^. A second research group has shown that increasing gel substrate stiffness above ~30 kPa correlates with increases in the speed of TFP-driven twitching in *P. aeruginosa*^22^. However, using the same *PaQa* reporter, intracellular signaling was not found to correlate with changes in substrate gel compositions (*i.e*. changed stiffness)^22^. These findings imply that surface mechanics seem to affect bacteria behaviors in a complicated way, and we therefore need a better understanding of how bacteria perceive and respond to the mechanics of surfaces where they attach.

The initial accumulation of bacteria on surfaces generally lead to biofilm formation. Passive adhesion of bacteria (and other colloids) will be strongly impacted by the surface energy and how this is reduced by adhesion, to reduce the system’s free energy. The hydrophobicity and hydrophilicity of surfaces can be major contributors to surface energy and thereby impact bacterial adhesion^23–27^. The trend of adhesion on hydrophobic and hydrophilic surfaces is different^28^. Electrostatics of the surface and the liquid medium, as well as van der Waals forces between bacteria and the surface, can also impact bacterial adhesion^26,27,29–31^. Living bacteria are not, of course, passive colloids, and may have the biological ability to respond actively to surface properties other than surface energy.

Some research has shown that the accumulation of bacteria varied on surfaces with different mechanics^32–35^. These earlier studies changed surface elasticity by varying characteristics such as cross-linking density or polymer concentration. Sometimes, an inappropriate fabrication may introduce unintended changes to other surface properties, resulting in unintended changes in surface energy, porosity, or the density of adhesion sites, and thereby impact adhesivity (see *Supplementary discussion*), which could act as a confounding factor to obscure the impact of surface elasticity. Perhaps as a result, the literature on the effect of surface mechanics on bacterial accumulation on surfaces does not show consistent trends^32,33,35^. Furthermore, many questions remain about the processes underlying how bacteria modulate their accumulation in response to surface mechanics. These questions are of critical importance because they prevent the design of strategies for controlling early biofilm development by manipulating surface mechanics.

To address these questions, in the present study, we used thin and thick hydrogels coated on glass coverslips to create composite materials with the same gel type, adhesivity and surface chemistry, but with different effective stiffnesses, and monitored *P. aeruginosa* through the early stages of biofilm formation on these surfaces. First, surfaces were exposed to a suspension of bacteria for one hour before quantitative microscopy was used to measure bacterial accumulation. For the immediately-following stages of biofilm formation, characterized by bacteria reproducing on a surface rather than accumulating on the surface from a suspended (planktonic) population, we measured the duration and growth rate of the biofilm lag phase and exponential growth phase, respectively.

For both the accumulation and reproduction stages of biofilm development, we show that bacteria actively recognize and respond to surface mechanics. When bacteria initially attach to a surface, both finite element modeling and experimental measurements of the activity of mechanosensitive ion channels show that attachment to thin composites causes greater changes in the mechanical stress and strain state of the bacterial cell envelopes than does attachment to thick composite. We also find that attachment to thin composites results in higher levels of intracellular c-di-GMP, which leads to greater reduction in motility, a reduced likelihood of detachment, and, as a result, greater accumulation on thin composites. Once the initial accumulation stage has passed, higher levels of cyclic-di-GMP are associated with a longer biofilm lag phase on thin composites. Modulation of c-di-GMP levels in response to surface mechanics is mediated by the cell-surface-exposed protein PilY1, a proposed mechanosensor. In short, this work uses a combination of several imaging modalities, quantitative image analysis, and physical modelling to advance our understanding of the mechanism(s) of mechanical signaling for an important human pathogen.

## Results

### Adhesion to thin composites leads to greater changes in mechanical stress and strain in the bacterial cell envelope than does adhesion to thick composites

**Fig. 1:**
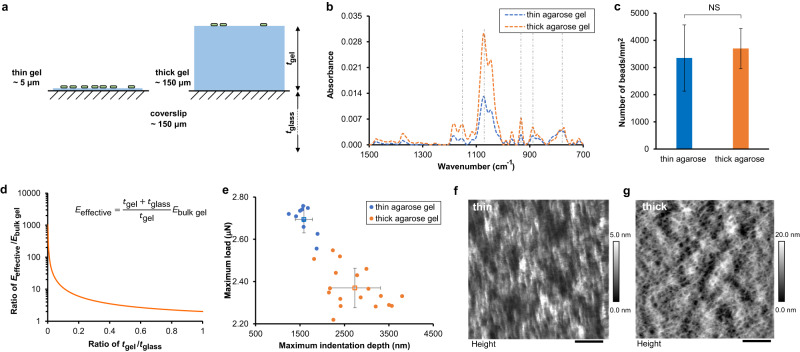
Fabrication of thin gel and thick gel-coverslip composites with different surface mechanics. **a** Schematic illustration of composites with different thicknesses of hydrogel, *t*_gel_, on top of glass coverslips with constant thickness *t*_glass_. **b** FTIR spectra of agarose gel composites with two thicknesses. The dash-dot lines indicate the location of characteristic peaks. *N* = 3. **c** The number of beads attached on agarose gel composites after incubation with bead suspension for 1 h. NS, not significant (*P* = 0.15). ANOVA test. NS indicates that the attachment of beads on thin and on thick gels are not significantly different for agarose gel composites. Data are means ± SD. *N* = 2. **d** The effective Young’s modulus of the hydrogel-coverslip composite (*E*_effective_), where *E*_bulk gel_ is the modulus of bulk hydrogel. The Young’s modulus of bulk agarose (3%) gel reported in Kolewe et al. work^32^ was 44.8 kPa. The calculated effective composite moduli of thin and thick agarose gel and glass composites are 1388.8 and 89.6 kPa, respectively. The Young’s modulus of bulk alginate (2%, 50 mM CaCl_2_) gel reported in Nunamaker et al. work^89^ was 32.0 kPa. The calculated effective composite moduli of thin and thick alginate gel and glass are 992.0 and 64.0 kPa, respectively. **e** Nanoindentation results of agarose gel samples. The relation between maximum load and maximum indentation of indentation curves of gels subjected to large indentation. Individual measurements are shown with solid circles; the means for all measurements per gel type are shown with hollow squares. Error bars are standard deviations. **f, g** AFM images showing the surface topography of thin and thick agarose hydrogels. Scale bar in **(f)**: 800 nm. Scale bar in **(g)**: 1 μm. Greyscale map of height is indicated to the right of each panel.

To eliminate effects arising from physicochemical properties of surfaces other than elasticity, such as adhesivity and surface chemistry, we used the same gel composition to fabricate thin and thick hydrogels atop glass coverslips (Fig. 1a and Supplementary Fig. 1a). Thickness measurements, done in triplicate for each hydrogel and thickness combination, found that thin gels were ~5 μm in height and thick gels were ~150 μm in height. We chose this geometry-based approach to modifying substrate mechanics to avoid inadvertently altering material adhesivity/chemistry along with mechanics, which has been observed before (Supplementary discussion) - for instance, poly(dimethylsiloxane) (PDMS) can have different surface adhesivities associated with different stiffnesses, shown by polymer beads found to accumulate more on soft PDMS than on stiff PDMS^36^. Using Fourier-Transform Infrared Spectroscopy, we confirmed that the surface chemistry of thick and thin composites was the same (Fig. 1b and Supplementary Fig. 1b). To confirm that the composite materials had the same surface adhesivity regardless of gel thickness, we incubated both thin and thick hydrogel composites with a suspension of fluorescent polystyrene polymer beads for one hour, and imaged the number of beads attached using confocal microscopy. We verified that the numbers of polystyrene beads that attached did not significantly differ with hydrogel thickness (Fig. 1c and Supplementary Fig. 1c). Thus, we conclude that hydrogel thickness does not impact passive physicochemical adhesion to surfaces.

However, the thickness of the hydrogel coated onto rigid glass coverslips does impact the mechanics of the resulting composite material. Linear elasticity theory was used to derive a closed-form expression for the effective elastic modulus (*E*_effective_) of hydrogel-coverslip composites (Supplementary Discussion and Supplementary Equation 7). For a hydrogel thickness (*t*_gel_) comparable to the 1 micron size of a bacterium, the *E*_effective_ increases sharply with decreasing *t*_gel_ (Fig. 1d). According to this model, the composites with thin (~ 5 μm) hydrogels are approximately 16 times stiffer than those with thick (~ 150 μm) – effective composite moduli are calculated at ~1 MPa for thin composites and ~50 kPa for thick composites (Fig. 1d and Supplementary Table 1 footnote). We also used a nanoindenter to experimentally impose loads on both types of composites that achieved indentation depth that was 1 μm (comparable to bacterial size) or greater (Fig. 1e). The indentation for a given force and tip geometry was consistently less for the thin gel than for the thick gel, with the maximum indentation depth to maximum load ratio being ~125 nm/μN for thick composites and ~75 nm/μN for thin composites. This experimentally validates that the composite made with thin gel is stiffer than the composite made with thick gel (Supplementary discussion and Fig. 1e).

Cryo-electron microscopy showed no discernible difference between the surface topographies of thick and thin gels (Supplementary Fig. 1d, e). However, atomic force microscopy (AFM) shows slight differences in the topographies, with measured average roughness of 1.25 nm for thin agarose gels and 2.62 nm for thick agarose gels (Fig. 1f, g). This ~1 nm difference in roughness is 3 orders of magnitude smaller than 1 μm bacteria, and this size discrepancy makes it unlikely that differences in surface topography could be sensed by bacteria. Moreover, others have found that the virulence response of *P. aeruginosa* upon adhesion is dependent on the stiffness of the surface but not related to the sizes of surface pores ranging from less than 10 to more than 1000 nms^21^. AFM measures an average roughness of 6.2 nm for thin alginate gels and 9.5 nm for thick alginate gels (Supplementary Fig. 1f, g). This ~3 nm difference in roughness is similarly far too small for any impact on bacteria to be expected.

Next, we sought to evaluate the degree to which the mechanics of composite substrates impacts bacteria. Upon surface attachment, bacteria are subjected to a mechanical tension in their inner membranes arising from nanoscopic cell envelope deformation due to the adhesion force exerted by the surface^37–39^. If topography had any effect on bacteria, we would expect that the rougher thick composite would exert a greater net adhesion force, due to the increase in surface area consequent to greater roughness. To test whether we could observe a difference in the membrane tension between bacteria adhering to the thin and to the thick composites, we compared the membrane tension in bacteria attached to both composites by measuring the activity of mechanosensitive ion channels. Other researchers have already established much about the mechanical activation of these ion channels – here, our focus is not to understand the ion channels themselves but rather to use their response as a readout to assess whether bacteria experience different degrees of mechanical change when they attach to thin composites than when they attach to thick composites.

These channels are located on the inner, cytoplasmic membrane^40^ and act as transducers of membrane tension - closed when the membrane is at low tension and open when the membrane is at high tension, allowing ions to pass through^40,41^. The two major mechanosensitive ion channels are large-conductance- and small-conductance- (MscL- and MscS-, respectively) type channels. When open under increased membrane tension, these channels provide non-selective pores of large and small diameter, respectively, through which sodium ions, Na^+^, can pass in very similar ways^41^. We pre-loaded bacteria with a fluorescent indicator for Na^+^ and then allowed them to sit for one hour attached to thin and thick agarose gels, in the presence of excess external Na^+^, before measuring the indicator brightness distribution as a proxy for internal Na^+^ levels.

**Fig. 2:**
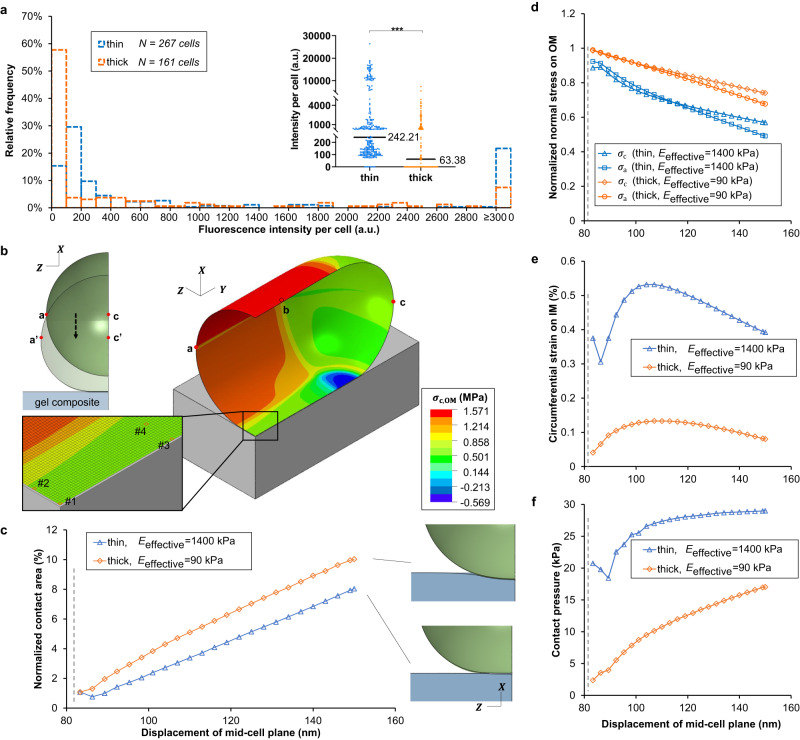
Adhesion to a thin gel surface leads to greater changes in mechanical stress/strain in the bacterial envelope and increased permeation of the bacterial cell membrane by sodium. **a** The histogram shows the average intracellular fluorescence intensity per cell of attached WT on thin and thick agarose gel composites after incubating with surfaces for one hour. Inset: Dot plot of the histogram, shown with median values. ^***^P < 0.001; Mann-Whitney u test. This indicates a statistically-significant difference between fluorescence intensity distributions and between median fluorescent intensities for cells on thin and thick gel composites. *N* = 3. The number of cells analyzed for each replicate are 73, 104, and 90 cells in the experiments on thin gels, and 62, 31, and 68 cells in the experiments on thick gels. **b** The finite element model and schematic illustration. Displacement along –X coordinate is applied on curve abc to bring the cell into contact with the surface. The heat map denotes the circumferential stress on OM (outer membrane). Inset: The representative elements analyzed in this study. **c** Contact area with different degree of indentation (displacement along –X coordinate). Contact area is normalized to the cellular surface area in the undeformed configuration. The dash line denotes when the cell first contacts the surface. **d–f** OM stresses become less tensile whereas IM (inner membrane) strain increases at element #1 upon surface adhesion. The degree of changes is greater on thin gels. Contact pressure is greater on thin gels. Subscript c denotes the circumferential direction and subscript a denotes the axial direction. Stresses are normalized to their respective values during the free-floating state and strains are the net change with respect to their respective values during the free-floating state.

The brightness distribution for bacteria on the thin composites had a peak at 100–200 arbitrary units (a.u.), whereas the brightness distribution for bacteria on thick composites had a peak, representing more than 60% of cells, at 0 to 100 a.u. (Fig. 2a). Both the median (Fig. 2a inset) and the mean fluorescence intensity of bacteria on thin composites were significantly greater than that of cells on thick ones – cells on thin gels had a mean fluorescence intensity of 2840.70 a.u. [2217.75 a.u., 3463.65 a.u.] (95% confidence interval) and cells on thick gels had a mean fluorescence intensity of 677.97 a.u. [478.28 a.u., 877.67 a.u.] (95% confidence interval). These results show that bacteria on thin composites are more permeable to Na^+^ than are bacteria on thick composites. Since mechanosensitive ion channels increase permeability upon increased membrane tension, we interpret this finding as indicating that bacteria have higher membrane tension when attached to thin gel surfaces than to the thick. *This is the opposite of what we should expect if the measured differences in surface topography were impacting bacteria*. However, it is entirely congruent with what we should expect if differences in composite mechanics were impacting bacteria, as follows:

Adhesive forces will tend to increase the area of the bacterium in contact with the surface, by deforming the bacterium and the surface. The energy costs for deforming the bacterium and the surface will depend on the elasticity of each. Mechanical equilibrium will be found by minimizing the sum of elastic energy costs (from cell and substrate deformation) and the adhesive energy benefit (from contacting area). Therefore, for constant adhesive area and bacterial elasticity, we expect that the deformation of the bacterial cell envelope will depend on the elasticity of the substrate (i.e., surface mechanics). To validate the trends shown by our experiment results and to elucidate further the relationship between surface mechanics and mechanical stresses in adhering bacteria, we developed finite element models (Fig. 2b and Supplementary Fig. 3) to simulate bacterial attachment to gel-coverslip composites. At the molecular level, bacterial surface properties and how they impact attachment to substrates are complex and not well-known^42^. Therefore, we approximated the adhesion process by displacing bacteria into contact with surfaces (Supplementary Discussion, Supplementary Fig. 3c, d). Using our models, we compared the bacterial envelope mechanics for bacteria interacting with thin and thick gel surfaces for a range of contact-increasing displacements. For any given displacement, the total contact area is greater for bacteria on the thick gel than on the thin one (Fig. 2c), reflecting the fact that the energy cost for deforming a soft material is lower than the cost for deforming a stiff one by the same amount. The initial, free-floating cells were subjected only to a turgor pressure (biologically, this arises from the osmolarity difference between the cytoplasm and the exterior), so that bacteria were in a pre-stressed state. Contact with a surface leads to a decrease in membrane stresses on the outer membrane, an increase in circumferential strain on the inner membrane, and the development of contact pressure (Fig. 2d and Supplementary Fig. 4a–i). These changes are all more pronounced when bacteria attached to thin gel surfaces.

Thus, our modeling results, showing greater strain in the bacterial inner membrane when attached to thin composites than to thick, are consistent with experimental measurements of the activity of mechanosensitive ion channels being greater on thin composites than on thick. This confirms that adhesion to thin gel and glass composites causes greater changes in the mechanical state of the bacterial envelope than does adhesion to thick gel and glass composites, indicating that this effect arises from different composite mechanics. Therefore, the most reasonable expectation is that any difference in bacterial response to the two types of composites should be linked to the difference in effective elasticity of the thick and thin composites.

### Early steps in biofilm initiation

The formation of a biofilm on a surface begins when bacteria initially encounter and attach to a surface. Bacteria can either then remain on the surface or detach back into the free-swimming, planktonic phase. The total *accumulation* of bacteria on a surface will depend on both the rate of attachment and the rate of detachment. Subsequent to this initial accumulation (and assuming that the reservoir of planktonic bacteria is removed), the population of bacteria on the surface will experience a phase of little change. This is known as the biofilm *lag phase*, and results from a combination of bacteria replication and detachment from surfaces, such that the population of surface-bound bacteria does not increase^43,44^. The lag phase ends with the onset of *exponential growth* of the population of surface-bound bacteria. Having established in the preceding subsection that adhesion to substrates with different composite elasticities results in different mechanical changes in the bacterial cell envelope, the remainder of our study here focuses primarily on the accumulation of bacteria on surfaces with different composite elasticities, and secondarily on the lag phase and exponential growth of bacteria on surfaces with different composite elasticities.

The pore sizes of the agarose and alginate hydrogels used are far too small to allow the bacterial body to enter the hydrogel; this is confirmed by microscopy observation that all sessile bacteria are attached to the top hydrogel surface, at the gel-fluid interface. This study focuses solely on bacteria attached to the hydrogel surface, and not on any free-swimming, planktonic bacteria in the fluid phase above. This is appropriate to our goal of better characterizing the role of bacterial mechanosensing in early biofilm development. Since the hydrogel thickness does not notably impinge on surface adhesivity or other surface properties (Fig. 1b, c, f, g and Supplementary Fig. 1b–g), the mechanosensing investigated here is that arising from substrate elasticity. Different adhesion forces^45^ and differences in the stiffness of bacterial cell walls^37^ could also give rise to different mechanical changes in bacterial envelopes upon adhesion, but these two parameters are not varied in the present study.

### PilY1 allows *P. aeruginosa* to differentially accumulate on thick and thin composites

**Fig. 3:**
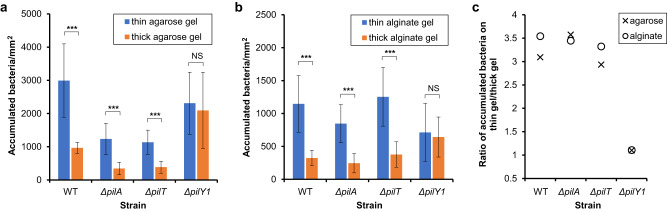
More bacteria accumulate on thin gel composites during one hour’s incubation for initial attachment. **a** The accumulation of WT, *∆pilA*, *∆pilT* and *∆pilY1* on thin and thick agarose hydrogel composites was determined after incubating with surfaces for one hour. **b** The accumulation of bacteria on thin and thick alginate hydrogel composites. Data are means ± SD. ^***^P < 0.001; NS, not significant (*P* = 0.28 for agarose; *P* = 0.29 for alginate); analysis of variance (ANOVA) test. **c** The ratio of accumulated bacteria on thin to that on thick hydrogel composites. These measurements were done using phase contrast microscopy for *N* = 4 replicates in all cases. Each replicate was imaged with at least 12 randomly-chosen fields of view.

As mentioned above, two categories of sensing pathways may be involved in the mechanosensing by *P. aeruginosa*. Therefore, to assess the impact of surface mechanics on the accumulation of bacteria on surfaces, we incubated the bacterial suspension of wild-type cells (WT), mutants without TFP (*∆pilA*), mutants without the PilT retraction motor (*∆pilT*) and mutants without PilY1 (*∆pilY1*) for one hour with hydrogel-coverslip composites and measured the bacterial accumulation on these surfaces by visualizing the number of bacteria using phase contrast microscopy. Consistent with some previous reports^32–34^ but not with others^35^, WT accumulated significantly more on thin composites than on thick composites (Fig. 3a, b), as did the *∆pilA* and *∆pilT* mutants (Fig. 3a, b). These findings were true across two types of hydrogels, agarose and alginate, supporting the idea that the difference in accumulation is likely to arise from the ~16x contrast in composite elasticity rather than details of surface chemistry.

Accumulation on the thin composites was greater by a factor of ~3.3 for all three strains (Fig. 3c). Thus, while functional TFP can increase the “baseline” accumulation, they have no measurable impact on the greater likelihood of accumulating on thin composites. Similar effects were found for the more starkly-contrasting case of glass versus agarose gel surfaces (Supplementary Discussion and Supplementary Fig. 2a).

In contrast, *∆pilY1* accumulated equally on thin and thick composites (Fig. 3a, b); this also is true for both agarose and alginate gels. This indicates that *P. aeruginosa* requires the cell-surface-exposed protein PilY1 for distinguishing between, and responding to, different surface mechanics. Again, similar effects were found for the more starkly-contrasting case of glass versus agarose gel surfaces, implying a much more muted response to stiffness difference by *∆pilY1* (Supplementary Discussion and Supplementary Fig. 2a). Adhesion-induced changes can only happen following, not preceding, bacterial contact with surfaces. Since gel thickness does not impact physiochemical surface adhesivity (Fig. 1c and Supplementary Fig. 1c), we expect bacteria to have equal likelihood of encountering and initially sticking to thin and thick composites. Therefore, this finding shows that greater accumulation on thin composites must arise as the result of something that happens after initial surface engagement – i.e., there is an active bacterial response to surface mechanics.

Thus, we hypothesize that WT initially adhered to thick composites will be more likely to detach than cells initially adhered to thin composites and that this difference should require PilY1. We test this hypothesis below.

### PilY1 mediates flagellar spinning and detachment rate in response to surface mechanics

**Fig. 4:**
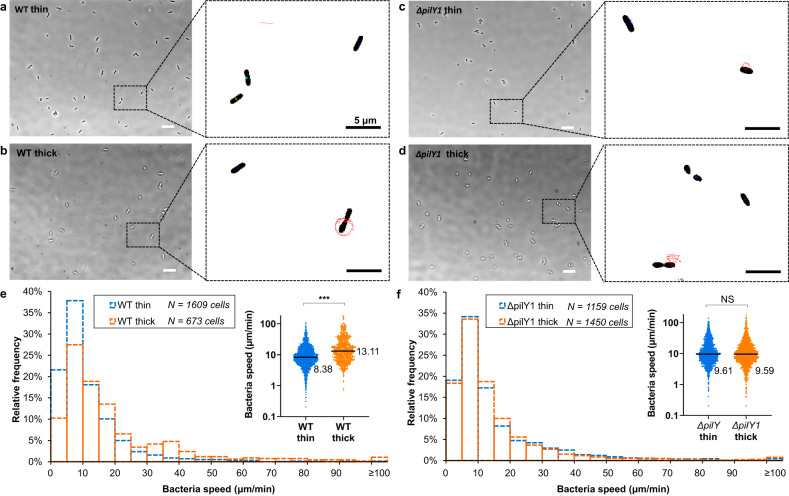
Adhered bacteria spin during the first hour of accumulation. **a–d** Phase contrast images of WT and the *∆pilY1* mutant adhered to thin and thick agarose gel composites. Insets: Tracked trajectories of bacterial centers-of-mass over 62.6 s. Scale bar: 10 μm. **e, f** Histograms showing speed distributions of WT and the *∆pilY1* mutant on thin and thick gel composites. Insets: Dot plots of the corresponding histogram. The median value is written to the right of each plot. ^***^P < 0.001; Mann–Whitney u test. *** indicates a statistically-significant difference in the distributions of WT speeds on thin and on thick gel composites and that the median speed of WT adhered to thick gel composites was higher, with statistical significance, than that of WT to thin gel composites. In contrast, NS (not significant) indicates that there is no statistically-significant difference in the distributions of speeds or in the median speeds of the *∆pilY1* mutant on the two composite types (*P* = 0.66, Mann–Whitney u test). Biological replicates *N* = 3 in all cases, with each biological replicate represented by 15 video sequences at randomly-chosen fields of view.

Shortly after encountering a surface, many *P. aeruginosa* cells are reversibly tethered by their flagella, which drive spinning about the surface-attached portion of the flagellum (to optical microscopy, the axis of rotation usually appears to go through one end of the cell). Spinning facilitates detachment from surfaces^46,47^. A deficiency in spinning is also associated with decreased probability of detachment^46^. Bacteria can also use TFP to move laterally on surfaces, but, during the first hour after bacteria were introduced to hydrogels (i.e., what we have termed the accumulation stage), we found that the vast majority of surface motility was in the form of spinning (Supplementary Fig. 5a, b). Therefore, we tracked the center-of-mass speed of surface-adhered bacteria (Fig. 4a–d) as a measure of spinning motility. We expect that a population with faster-spinning bacteria will have a higher rate of detachment^48^.

For WT, the distribution of spinning speeds on thick composites was much broader than thin composites (Fig. 4e). Both the median (Fig. 4e inset) and the mean speeds on thick composites were significantly higher than on thin composites - mean speed of 20.06 μm/min [18.43 μm/min, 21.68 μm/min] (95% confidence interval) on thick composites and mean speed of 11.46 μm/min [10.95 μm/min, 11.97 μm/min] (95% confidence interval) on thin composites. In summary, WT are more likely to spin rapidly on thick composites than on thin composites. Upon tracking cells, we indeed found that WT were significantly more likely to detach from thick gels (30 detachment events among 673 tracked cells) than from thin gels (10 detachment events among 1609 tracked cells) (*P* < 0.001, χ^2^ test) (Supplementary Fig. 5c). This is an active bacterial response to surface mechanics.

For the *∆pilY1* mutant, the peak spinning speed was unchanged from that of WT (Fig. 4e, f), suggesting that loss of PilY1 does not intrinsically disrupt spinning motility. However, for the *∆pilY1* mutant, neither the distributions of spinning speeds nor the median spinning speeds were significantly different on thin and thick composites (Fig. 4f). The mean speed was 15.08 μm/min [14.16 μm/min, 16.01 μm/min] (95% confidence interval) on thin composites and 14.86 μm/min [13.92 μm/min, 15.81 μm/min] (95% confidence interval) on thick composites. Furthermore, the *∆pilY1* mutant was equally likely to detach from thin and thick composites (*P* = 0.78, χ^2^ test) (Supplementary Fig. 5c). These results are strikingly unlike those for WT and imply that *P. aeruginosa* lacking PilY1 do not adjust their spinning motility, and therefore their likelihood of detachment, in response to surface mechanics. PilY1 is linked to regulating flagellar activity either up or down - increasing spinning speed on thick composites and decreasing spinning speed on thin composites (Supplementary Fig. 5d,e). Notably, we find a linear correlation between spinning speed and the probability of detachment (Supplementary Fig. 5d,e)

These findings raise the question of what provides the causative linkage between PilY1 and changes in flagellar activity. A key regulator of flagellar motility in *P. aeruginosa* and many other microbes is the intracellular second messenger cyclic diguanylate (c-di-GMP)^49,50^. We have shown that, compared with thick composites, thin composites could lead to higher membrane tension in the adhering bacterium. In addition, others have shown that the unfolding and misfolding of inner-membrane and periplasmic proteins associated with surface attachment results in an elevated level of c-di-GMP for *P. aeruginosa*^12^.

Therefore, we hypothesized that different surface mechanics arising from thin and thick composites would cause different levels of c-di-GMP production upon surface attachment, and that PilY1 is involved in the c-di-GMP response. We test this hypothesis below.

### Composite mechanics impact c-di-GMP signaling in a PilY1-dependent manner during bacterial accumulation

To see whether PilY1 modulates c-di-GMP dynamics in response to composite mechanics, we used a validated reporter plasmid, P_*cdrA*_::*gfp*, that produces green fluorescent protein (GFP) in response to increases in c-di-GMP^51^. We previously used this plasmid study c-di-GMP signaling in bacterial mechanosensing of shear^13^. We measure average per-cell GFP intensity, accounting for attenuation by the substrates, as we did in earlier studies^13,52^.

**Fig. 5:**
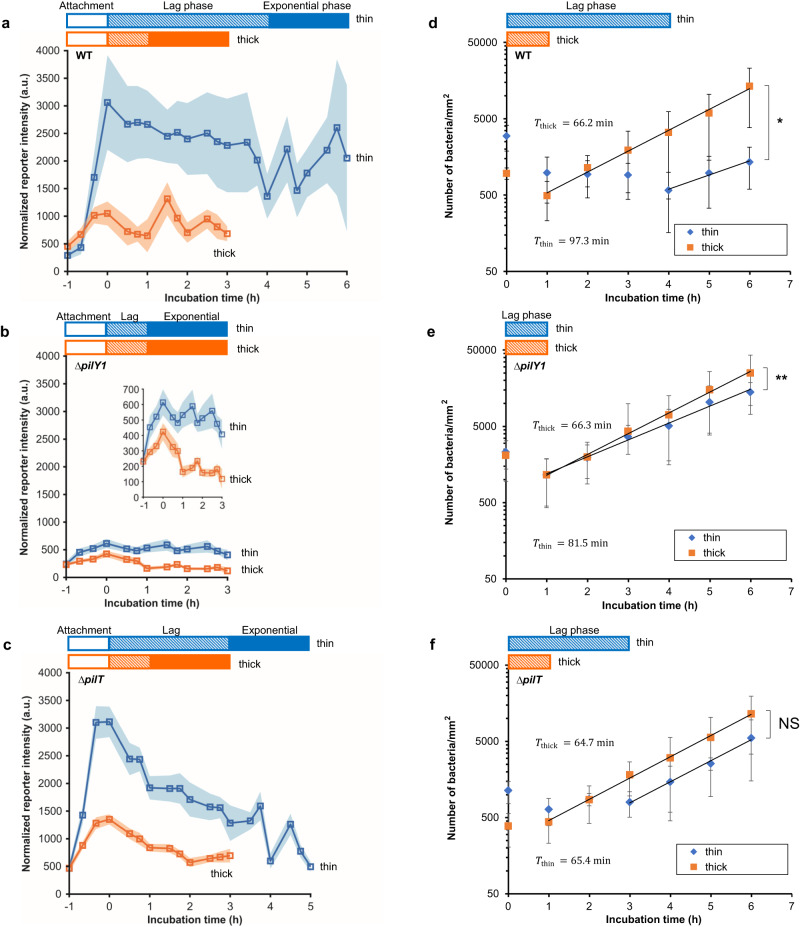
On agarose surfaces with different mechanics, PilY1 acts to mediate the duration of the lag phase in biofilm growth and the levels of the intracellular signal c-di-GMP, and PilT is required to mediate the growth rate of the exponential phase of biofilm growth. **a–c** The average per-cell normalized intensity for fluorescent reporters for changes in intracellular c-di-GMP in WT and the *∆pilY1* and *∆pilT* mutants during accumulation, lag phase, and exponential phase. The same vertical scale is used for each plot so that differences between strains are clear. The initial hour of accumulation on a surface is designated by −1 to 0 h, shown by hollow color bars. For each sample, exponential phase was observed for two hours, shown by solid color bars. Squares represent mean levels of c-di-GMP at each time point, linked by lines as a guide to the eye. Shaded regions correspond to 95% confidence intervals. The inset in (**b**) shows c-di-GMP reporter intensity in the *∆pilY1* mutant with a smaller y-axis range. *N* = 3 for all experiments using the reporter plasmid; *N* = 2 for all experiments using the control plasmid. **d–f** Growth dynamics of attached WT, and the *∆pilY1* and *∆pilT* mutants on thin and thick agarose gel composites. Data are means ± SD. The data at 0 time point corresponds to the end of one hour of bacterial accumulation on gel surfaces. The accumulation phase was always one hour long, and was set by the time that a suspension of planktonic bacteria was incubated with the surface. Hatched color bars show the length of the lag phase. The duration of the lag phase was experimentally determined in each case, by measuring the bacterial population on the surface. While that population was roughly constant in time, the system was considered to be in lag phase. The onset of exponential growth phase was determined experimentally in each case, by measuring the bacterial population on the surface. Once the population started to increase as an exponential function of time, the system was considered to be in exponential phase. The doubling time, *T*, is calculated by the equation *T* = ln2/*α*, where *α* is the growth rate of bacteria on surfaces (equations of exponential regression, f(*t*) = A*e*^*αt*^, where *t* is the incubation time). For each bacterial strain, we use T_thick_ to designate the doubling time on the thick gel composite, and T_thin_ to designate the doubling time on the thin gel composite. ^**^P < 0.01, ^*^P < 0.05; NS, not significant; analysis of covariance (ANCOVA) test. ** and * indicate that the growth rate *α*_thin_ is significantly different from *α*_thick_ for WT and for the *∆pilY1* mutant, while NS means the difference in growth rates on thin and thick gel composites are not significant for *∆pilT* (*P* > 0.1). Each time point was done for two replicate samples, and at least 12 fields of view were randomly chosen for each replicate. Samples used for measurement at one time point were not used for further incubation or later measurements, i.e., the measurement at each time point was done independently. Thus, for each strain and thickness combination, 14 replicas were measured.

For bacteria containing PilY1, we found a sharp rise in c-di-GMP levels during the initial hour of accumulation (−1 to 0 h in Fig. 5a), which is consistent with previous findings that c-di-GMP levels in *P. aeruginosa* increase upon surface attachment^9,13,53^. At the end of the “accumulation” hour (i.e., the beginning of the incubation time), WT on thin composites had significantly higher c-di-GMP levels than did WT on thick composites. This meshes with our finding that WT on thin composites had lower spinning motility than those on thick composites (Fig. 4e), as high levels of c-di-GMP inhibit bacterial motility^49^. These data also suggest that the causative linkage between PilY1 and changes in flagellar activity (which, in turn, modulate the likelihood of detaching from the surface), is likely via PilY1-controlled c-di-GMP signaling.

On both thin and thick composites, the *∆pilY1* mutant had much lower c-di-GMP levels than did WT (Fig. 5a, b); this finding is consistent with the role of PilY1 in regulating c-di-GMP production^9^. For WT, the mean level of c-di-GMP at the end of the “accumulation” hour was ~2.9 times higher on thin composites than on thick composites, but it was only ~1.4 times higher for the *∆pilY1* mutant (Fig. 5a, b). This observation is consistent with a loss of the ability to discriminate surface mechanics. This finding is also consistent with the causative connection that PilY1 is required for bacterial mechanoresponse linking surface mechanics to c-di-GMP signaling levels during the initial “accumulation” phase.

This finding also raises the question of how PilY1, and consequent changes in c-di-GMP signaling, impact the growth of the bacterial population on the surface.

### PilY1 impacts biofilm growth in the lag phase, in response to composite mechanics, by modulating c-di-GMP signaling

When planktonic bacteria are introduced into new liquid medium, they experience a temporary period of non-replication, termed the “lag phase”^54^. After attachment to a glass surface, *P. aeruginosa* also undergoes a lag phase before exhibiting exponential growth^43,44^. However, unlike the planktonic lag phase, the lag phase of biofilm growth involves a combination of bacteria replication and detachment from surfaces, such that the population of surface-bound bacteria does not increase^43,44^.

After allowing bacteria to accumulate on the surfaces of composites for one hour, we replaced the bacterial suspension with fresh, sterile culture medium, so that no more bacteria can attach to the surface from the liquid phase. We designate this timepoint the beginning of the incubation time (0 h in Fig. 5). The duration of the lag phase, from the beginning of the incubation time to the onset of exponential growth, is given by the lag time, *τ*_lag_, indicated by hatched color bars in Fig. 5. WT populations had a *τ*_lag_ of 4 h on thin composites and 1 h on thick composites, but *∆pilY1* populations had the same *τ*_lag_ of 1 h on both composite types (Fig. 5d, e). Similar results were found for bacterial growth on bulk gels (soft) and glass slides (stiff) (Supplementary Fig. 2b, c). These results show that composite mechanics can markedly impact the growth of the sessile bacterial population, and that PilY1 is key for this process as well as for the accumulation preceding incubation. When PilY1 was complemented back on an arabinose-inducible plasmid, the *∆pilY1* mutant populations again had different *τ*_lag_ on thin and thick composites (Supplementary Discussion and Supplementary Fig. 6c), confirming PilY1’s role in surface mechanics sensing. Interestingly, although the early c-di-GMP response of *∆pilT* was indistinguishable from that of WT, during the subsequent incubation phase the c-di-GMP levels of *∆pilT* dropped much more quickly than they did for WT (Fig. 5c).

On both thin and thick gels, c-di-GMP levels in WT fell during the lag phase and subsequently oscillated once populations entered the exponential growth phase (Fig. 5a). The high level of c-di-GMP induced by the initial mechanical stimulus of surface contact (0 h in Fig. 5a) allows bacteria to sense the surface and initiate a sessile lifestyle. However, it would be a metabolic burden for cells to maintain such high c-di-GMP levels in the following biofilm development. We speculate that bacteria may have to decrease the c-di-GMP level to allow the beginning of exponential biofilm growth on surfaces; this speculation is consistent with the work of others^55,56^. If so, the longer *τ*_lag_ for WT on thin composites than on thick composites likely arises from the much higher initial c-di-GMP levels on thin composites and the consequent need for more time to gradually decrease c-di-GMP levels. For *∆pilY1*, low initial levels of c-di-GMP are associated with a short *τ*_lag_ on both thin and thick composites (Fig. 5b, e). Interestingly, the lag phase of *∆pilT* on thin gels was slightly shorter than that of WT, and the *∆pilT* population’s rate of exponential growth is indistinguishable on the two composite types (Fig. 5c, f) – this is unlike the cases for WT and for *∆pilY1* populations, which have higher exponential growth rates on thick composites (Fig. 5d, e).

We conclude that PilY1 is a required element for controlling *P. aeruginosa*’s initial c-di-GMP response to surface mechanics and consequent lag time in early biofilm growth.

## Discussion

Our experimental results show that PilY1 may act as a signal amplifier that mediates c-di-GMP levels and flagellar motility in response to surface mechanics. It is also possible that PilY1 acts as a mechanosensor that transduces mechanical changes upon surface engagement into c-di-GMP signaling. PilY1 is a surface-exposed protein found associated with the TFP tip^9^, so PilY1 may be responding to the compressive loading incurred due to surface adhesion, a stress state identified in the modeling. A recent study suggested that the conformational changes of PilY1 lead to stimulation of bacterial c-di-GMP production and biofilm formation^57^. The compressive loading may hence engender the required conformational changes on PilY1 for biofilm initiation, while our modeling shows that bacteria adhered to thin composites surfaces will have a greater decrease in the tension in their outer membrane than will bacteria adhered to thick composites.

The differential response of mechanosensitive ion channels to surface mechanics (Fig. 2a) opens the possibility that mechanosensitive ion channels may play a role in the initial development in biofilms on surfaces, although we have not investigated that specifically. At the exponential-growth phase of biofilm formation, our data suggests that the pilus retraction motor PilT may also be involved in responding to surface stiffness in a way that modulates c-di-GMP level and growth rate (Fig. 5c, f)^58^; see Supplementary discussion.

The mechanical equilibrium of a system consisting of a bacterium adhering to a surface will be found when the net mechanical energy is minimized. Adhesion energy, which is energetically favorable and negative in sign, will increase in magnitude as the adhering area increases. Increasing the adhering area incurs elastic energy costs for deforming the bacterium and the surface; elastic energy costs are energetically disfavorable and positive in sign. More of the elastic energy cost will be borne by the bacterium when the surface is stiff than when it is soft. Therefore, for surfaces that have the same adhesive properties, bacteria adhering to thick composites will deform less than will bacteria adhering to thin composites; this has been confirmed by finite element modeling (Supplementary Fig. 3a, b) and by experiments measuring the activity of mechanosensitive ion channels (Fig. 2a).

For a given adhesion energy, stiffer bacteria would deform less and softer bacteria would deform more. This could alter the mechanosensing response to surface attachment. *P. aeruginosa* maintain tight genomic control of their stiffness^59^. This clearly has benefits for protecting the bacteria against mechanical stress, such as osmotic pressure. This may also benefit bacteria by safeguarding the surface-sensing response, which is essential to this biofilm-former.

The effective modulus of our composites with thin gel was roughly 1 MPa and the effective modulus of composites with thick gel was less than 100 kPa (Supplementary Table 1). These values bracket the stiffnesses reported for *P. aeruginosa* and other Gram-negative bacteria^60–62^. Gram-positive bacterial cells are stiffer^63^ than Gram-negative bacterial cells^64–67^. Bacteria themselves are a composite material, comprising the softer cytoplasmic interior and the stiffer envelope. The Young’s modulus for the envelope material per se of Gram-negative bacteria is roughly several tens of MPa, and the envelope material of Gram-positive bacteria probably has a similar modulus^64,68^. Our finite element modeling identifies bending as the major envelope deformation modality in the contact zone as bacteria attach to surface. According to the Kirchhoff-Love plate theory^69^, the flexural rigidity of a thin plate (effectively the modulus that measures the energy cost for bending a plate) is characterized by *Et*^3^/12(1−*v*^2^) α *t*^3^, where *E* is the Young’s modulus of the plate, *ν* is the Poisson’s ratio, and *t* is the plate thickness. Gram-negative bacteria have a much thinner peptidoglycan cell wall than do Gram-positive bacteria (the cell wall of *P. aeruginosa* (Gram-negative) is ~3 nm thick^70^ and that of *B. subtilis* (Gram-positive) is ~30 nm thick^71^). This observation suggests that Gram-positive bacteria will deform less than will Gram-negative bacteria upon adhesion to the same surface, because the energetic cost for deforming Gram-positive bacteria will be higher. Therefore, we suggest that Gram-positive bacteria may be less well-adapted to using envelope stress and strain to sense and respond to surface stiffness. This inference is in agreement with previous reports that Gram-positive bacteria do not respond to surface stiffness in the same way as Gram-negative bacteria^72,73^.

**Fig. 6:**
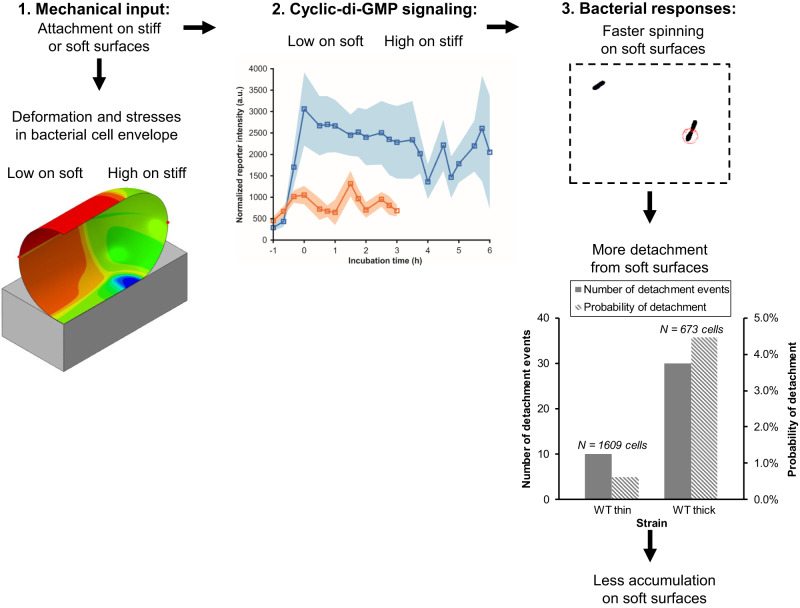
Schematic of the proposed links between surface mechanics and bacterial accumulation. Adhesion to a surface results in changes in mechanical stresses and deformations in the bacterial cell envelope. These changes are greater when the surface is stiff than when it is soft (Fig. 2). Adhesion to a stiff surface results in a greater increase in intracellular levels of c-di-GMP than does adhesion to a soft surface (Fig. 5). Higher levels of c-di-GMP result in faster bacterial spinning (Fig. 4) on soft surfaces and a greater likelihood of detaching from soft surfaces (Supplementary Fig. 5). As a result, more bacteria accumulate on stiff surfaces than on soft (Fig. 3).

In summary, in this study, we fabricated surfaces with different mechanics using thin and thick hydrogel and coverslip composites. Composite thickness does not change gel composition, surface chemistry (measured by FTIR), or surface adhesivity (measured by passive accumulation of beads). Therefore, these composites are well-suited to elucidating effect of surface mechanics on bacterial accumulation and growth, without many confounding factors arising from other passive physicochemical properties of surfaces^36^. We show that bacterial accumulation on surfaces strongly depends on substrate mechanics: Accumulation of bacteria on a surface is the first step leading toward biofilm development. Consistent with previous findings that misfolding and misregulation of envelope proteins causes elevated level of c-di-GMP for *P. aeruginosa*^12^, bacteria adhered to thin composites underwent a greater change in the mechanical strain and stress in the envelope than did bacteria adhered to thick composites. Adhesion to thin composites was also associated with higher c-di-GMP levels than was adhesion to thick composites. Higher c-di-GMP levels were the causative link that led to lower spinning motility, less detachment, and thus greater accumulation than did adhesion to thick composites. Figure 6 graphically summarizes these findings. The cell-surface-associated-protein PilY1 is required for this link between substrate mechanics and bacterial accumulation. Later biofilm growth also shows an impact of surface mechanics, PilY1, and PilT.

## Methods

### Strains and plasmids

We used *P. aeruginosa* PA01 WT and *ΔpilA*, *ΔpilT*, *ΔpilY1* mutants^74^. Studies of bacterial accumulation, motility, growth, and c-di-GMP production were done with bacteria that contained the plasmid P_CdrA_::*gfp*. This plasmid is a verified reporter for c-di-GMP; it is a transcriptional fusion between the cyclic di-GMP-responsive *cdrA* promoter and a gene encoding green fluorescent protein (GFP)^51^. Strains containing a promotorless control plasmid pMH487 were used to measure background GFP expression independent of c-di-GMP levels^13^. Confocal laser-scanning microscopy was used for measurements of GFP, with calibration for light attenuation by different gel composites^52^, and phase contrast microscopy (which imposes a lower light dose on bacteria and therefore reduces potential phototoxicity) was used to measure accumulation, motility, and growth. Strains with the P_CdrA_::*gfp* and pMH487 plasmids were grown with 60 μg/mL Gentamycin (Sigma-Aldrich, G1914) for plasmid selection. Fluorescence measurements using the sodium ion indicator were performed using WT that did not contain any plasmid.

### Fabrication of thick and thin agarose hydrogel composites

3% (w/w) agarose solution was prepared by dissolving agarose powder (Sigma-Aldrich, A9414) in Millipore water and then autoclaving at 121 °C for 15 min.

To form composites of thick agarose gel on glass coverslips, 20 μL of agarose solution was spread (while still liquid) inside an imaging spacer (Grace Bio-Labs, SS1X13, 13 mm diameter × 0.12 mm depth) adhered to a coverslip. This was cooled at 4 °C for 2 min in a humid chamber, resulting in gelation.

To form composites of thin agarose gel on glass coverslips, 3 μL of agarose solution (while still liquid) was pipetted onto a glass slide (prior to this, the glass slide had been sonicated in 70% ethanol (Pharmco, 111000200) for 15 min and then dried with nitrogen) and a coverslip was placed on top of the fluid drop. Two binder clips were used to clamp together the slide and coverslip, spreading the solution between them. After cooling at 4 °C for 2 min, the binder clips were removed and the coverslip was detached from the glass slide using a razor blade. This left thin agarose gel coated on the coverslip. The thick and thin agarose gels were then immediately immersed into buffer solutions or bacterial suspension for the following experiments.

### Fabrication of thick and thin alginate hydrogel composites

2% (w/w) sodium alginate (SA) solution was prepared by dissolving sodium alginate powder (Sigma-Aldrich, 180947) in Millipore water. After being stirred for 2 h, the SA solution was filter sterilized.

To make thick alginate gel, 20 μL of SA solution was spread into an imaging spacer (Grace Bio-Labs, SS1X13, 13 mm diameter × 0.12 mm depth) adhered to a coverslip that was pretreated with 500 μL of 0.1 mg/mL Poly-L-lysine (Sigma-Aldrich, P9155) for 2 h to facilitate adhesion of the alginate gel to the coverslip surface. The thick alginate gel was formed after the coverslip was immersed in 50 mM Calcium chloride (CaCl_2_, Sigma-Aldrich, C3306) solution for 2 h.

To make thin alginate gel, 1 μL of SA solution was pipetted onto a glass slide (the glass slide was sonicated in 70% ethanol for 15 min and then dried with nitrogen before use), and a coverslip that was pretreated with 500 μL of 0.1 mg/mL Poly-L-lysine for 2 h to facilitate adhesion of the alginate gel to the coverslip was placed on top of the SA solution. Following the spread of SA solution between the glass slide and the coverslip, CaCl_2_ solution was gently added along the sides of the coverslip and allowed to diffuse in for 2 h. The coverslip was removed from the glass slide using a razor blade, leaving the thin alginate gel coated on the coverslip.

Then both the thin and the thick alginate gel surfaces were immediately washed by Millipore water to remove extra CaCl_2_ before bacteria or beads were introduced.

### Measurement of thicknesses of hydrogels on coverslips

The thicknesses of hydrogels on coverslips were measured using an Olympus IX71 inverted phase contrast microscope with a 60× oil-immersion objective. Hydrogel composites were incubated with bacterial suspension (incubation details can be found in the following section). The microscope stage controller (Applied Scientific Instrumentation MS-2000) displayed the Z-positions of the stage when two locations were in focus (Supplementary Fig. 1a). These locations were a gel-free area at which bacteria were attached directly to the coverslip (Z_1_) and the top of the gel to which bacteria were attached (Z_2_). Taking the difference between Z_1_ and Z_2_ gave a measurement of the thickness of the gel on coverslips. Each experiment was repeated three times independently.

The 60x oil objective we used for these measurements has a numerical aperture of NA = 1.25. We have a filter in the microscope so that the illuminating light is green, with wavelength roughly *l* = 550 nm. The immersion oil used with this objective has a refractive index of about *n* = 1.5. Using the following approximation for depth of field^75^

1 $$d = \frac{{l\sqrt {n^2 - \left( {NA} \right)^2} }}{{\left( {NA^2} \right)}}$$

we obtain a depth of field of 291 nm. This is nearly an order of magnitude smaller than a 1-micron bacterial thickness, and more than an order of magnitude smaller than the 5-micron thickness we measure for the thin gel. Therefore, any uncertainties in the measured gel height arising from the depth of field of this microscope objective are negligible.

### Characterization of surface mechanical properties of hydrogel composites using nanoindentation

An Optics11 Life Piuma nanoindenter was used to perform surface mechanical measurements on agarose hydrogel composites. Measurements were performed on thick and thin composites (fabricated as described above) glued to the bottom of a petri dish and submerged in DI water at room temperature. A probe with cantilever stiffness of 0.25 N/m and a tip radius of 9.5 µm was used to determine the gel’s intrinsic Young’s modulus. A probe with cantilever stiffness of 0.23 N/m and a tip radius of 24 µm was used for assessing mechanical differences resulting from the composite structure (Supplementary Discussion). The probe loaded and unloaded samples at a constant piezo-motor displacement rate of 2 µm/s. All specimens were measured at 9 different locations near the center of the gel surface with two replicates for each gel thickness. Indentation profiles where the probe failed to make contact with the surface were excluded, resulting in at least 9 measurements per condition.

To determine the intrinsic Young’s modulus of a gel, a loading curve was fit with the Hertzian model^76^:

2 $$F = \frac{4}{3}\frac{E}{{1 - v^2}}R^{0.5}\delta ^{1.5}$$

where *F* is the load, *E* is the Young’s modulus, *R* is the tip radius, *δ* is the indentation depth, and *ν* is the Poisson’s ratio, which we assumed to be 0.5. Data were fit up to an indentation depth equal to 10% of the tip radius. The maximum load and maximum indentation of an indentation curve were recorded and used to examine the composite effect.

### Electron microscopy measurement of surface topography

For the purposes of Cryo Electron Microscopy, hydrogel samples were adhered to Electron Microscopy stubs using carbon tape. As suggested by the literature^77^ the samples were subsequently plunged into slush nitrogen for ten seconds to vitrify. The samples were immediately, whilst continuously held under vacuum, inserted into a FEI FEG XL30 microscope. For the purposes of the sublimation step, the sample temperature was raised from −170 °C to −90 °C before being lowered back down to −170 °C.

### AFM imaging and roughness measurements

AFM measurements were performed on a Bioscope Catalyst AFM (Bruker). AFM tapping mode was selected for these measurements to minimize the damage to the sample topography. Silicon tips with a resonance frequency around 300 kHz, 10 nm in radius, and a spring constant of 40 N were used. Measurements were performed under room temperature in aqueous conditions (Mili-Q water). For each AFM experiment a minimum of three different samples were investigated, and a representative height image of the surface morphology is reported in this article. The NanoScope software (Bruker) was used to analyze AFM images and to evaluate the film surface roughness of a 4 × 4 µm area from each sample. The mean roughness (R_a_) parameter used for these measurements, is the arithmetic average of the deviations from the center plane of the AFM image which is dependents on the sampling size.

### Characterization of surface chemistry of hydrogel composites using Fourier transform infrared spectroscopy (FTIR)

Composites of thin and thick agarose or alginate hydrogels on glass were prepared as described, except that this time thick hydrogels were coated on a glass slide instead of a coverslip; thin gels were still made on coverslips, as described above. Before mounting a hydrogel sample on the spectrometer, the imaging spacer that thick hydrogels were spread into was peeled off from a glass slide for firm contact between hydrogels and the ATR crystal of the spectrometer.

Infrared spectra were collected on a Bruker Vertex 70 Fourier transform infrared (FTIR) spectrometer equipped with a liquid nitrogen cooled HgCdTe (MCT) detector and a single reflection attenuated total reflectance (ATR) accessory consisting of a Ge ATR crystal with a 65° angle of incidence. Data were collected over the 400–4000 cm^−^^1^ range and manually cut to the 700–1500 cm^−^^1^ or 700–1900 cm^−^^1^ range for analysis. After hydrogel samples were mounted on the Ge ATR crystal, 500 scans at a resolution of 4 cm^−^^1^ and 20 kHz scan speed were accumulated to generate the single channel spectra for each sample. A blank spectrum of a clean Ge crystal was subtracted from the sample spectrum to generate the sample absorbance spectrum. A blank spectrum of a glass coverslip (for thin hydrogels) or glass slide (for thick hydrogels) was also subtracted. Finally, baseline tilt in the sample spectrum was corrected using the rubber band correction baseline function in the OPUS Spectroscopic software (OPUS, 6.5.92, Bruker Optik, GmbH, Ettlingen, Germany). Each combination of gel type and gel thickness were characterized in three replicate experiments.

### Measurement of bead attachment on surfaces

To compare the passive adhesivity of surfaces, we measured the passive attachment of polymer beads to the surface. For this, fluorescent polystyrene polymer beads (Bangs Laboratories, Inc., Dragon Green, FSDG004, diameter 1 μm) were diluted 1000 times in NaCl buffer (10 mM potassium phosphate composed of 5.4 mM potassium phosphate dibasic (K_2_HPO_4_, Sigma-Aldrich, 60353) and 4.6 mM potassium phosphate monobasic (KH_2_PO_4_, Sigma-Aldrich, 60218), 135 mM NaCl, pH 7.0).

16 μL of this bead suspension was added into one imaging spacer (Grace Bio-Labs, SS1X13, 13 mm diameter × 0.12 mm depth) adhered to a coverslip, which was then sealed by a hydrogel-coverslip composite (either thick or thin). After 1 h, beads attached on gels were imaged using an Olympus Fluoview1000 confocal microscope with a 60× oil-immersion objective. Fluorescent beads were illuminated with a 488 nm laser using standard GFP filter sets and confocal z-stacks were captured by Fluoview10-ASW version 4.2 software. The confocal z-stacks were processed using the particle analysis function in the Fiji distribution of ImageJ^78^ to quantitatively determine the numbers of beads attached on gel composites (i.e., areal density of beads (number of beads/mm^2^)). Each combination of gel type and gel thickness was tested in two replicate experiments and at least 5 fields of view were randomly chosen for each replicate.

### Microscopy measurement of bacterial accumulation on surfaces

Bacteria were streaked from frozen stock onto LB-Miller agar plates (Fisher Scientific, BP1425) and incubated overnight at 37 °C. Single colonies were inoculated into Luria broth (LB, 5 g of yeast extract (Fisher Scientific, BP1422), 10 g of tryptone (Fisher Scientific, BP1421), and 10 g of sodium chloride (NaCl, Sigma-Aldrich, S9888) per L of Millipore water) and grown overnight at 37 °C with shaking at 242 rpm using an orbital shaker (Labnet Orbit 1000). Then, 80 μL of overnight culture was transferred into 20 mL of fresh LB and vortexed. The resulting bacterial suspension was incubated at 37 °C with shaking at 242 rpm for at least 2 h until hydrogel composites were freshly made and ready. Then the bacterial suspension was incubated with hydrogel composites at 37 °C for 1 h.

Surface samples with accumulated bacteria were then gently washed with phosphate buffered saline (PBS, Sigma-Aldrich, P4417) twice, and then visualized using an Olympus IX71 inverted phase contrast microscope with a 60× oil-immersion objective. Images were taken by a QImaging EXi Blue CCD camera controlled by QCapture Pro-6 software and processed using the particle analysis function in Fiji to quantitatively determine the numbers of bacteria on surfaces (i.e., areal density of bacteria (number of bacteria/mm^2^)). For each combination of bacterial strain and hydrogel type (alginate or agarose), two technical replicates of each type of composite (thin and thick) were tested on each day (for a total of four samples per day). At least 12 fields of view were randomly chosen for each replicate. Each experiment was repeated twice independently on different days (for a total of 4 replicates for each combination of bacterial strain and composite type).

### Finite element modeling of the cell-surface interaction

Finite element models (ABAQUS/Standard 2021, Dassault Systems, Providence, RI, USA) were developed to simulate the structural interaction between bacteria and hydrogel substrates upon surface adhesion. A 3D model with geometric nonlinearity was created, and the quarter symmetry was utilized (*P. aeruginosa* are rod-shaped and substrates are cuboid, Fig. 2b). The simulations were performed on the Frontera Linux cluster of the Texas Advanced Computing Center^79^.

As a strain of Gram-negative bacteria, *P. aeruginosa* feature a thin layer of the bacterial cell envelope ( ~ 10^1^ nm^70^, compared to the whole cell ~10^3^ nm) that encloses the cytoplasm, chromosomes, and other intracellular materials. The bacterial cell envelope is a complicated multilayered structure composed of a rigid layer of cell wall sandwiched between two membranes made of lipid bilayers^80^. The membrane exterior to the cell wall is called the outer membrane (OM) and the membrane interior to the cell wall is the inner (or cytoplasmic) membrane (IM). Both OM and IM harbor a myriad of membrane proteins. The outer membrane is anchored to the cell wall via lipoproteins, and the outer membrane and cell wall together bear most of mechanical loading^62,81,82^. The high osmolarity difference between the internal bacterial cytoplasm and the external environment causes the bacterial cell envelope to be swollen by turgor pressure.

Recognizing these, bacteria were characterized as a thin-walled pressure vessel that consists of a hollow cylindrical trunk with hollow hemispherical caps on both ends with literature-reported properties (Supplementary Table 1). The envelopes were modeled as a two-layered composite material, the outer layer of which is the outer membrane and the inner layer of which is the cell wall. Hydrogel-coverslip composite substrates were assumed to be an isotropic and homogeneous material, with its properties computed from the composite theory (Fig. 1d) and the reported values (Supplementary Table 1).

Due to the thinness of bacterial cell envelopes, we used shell elements (24257 S4R elements with enhanced hourglass control, Supplementary Fig. 3a) to discretize them. The composite shell scheme was used to section cell envelopes into two layers of different materials, with three section points on each layer (at which secondary variables, e.g., stress and strain, were computed). Substrates were discretized by eight-node linear brick elements (436428 C3D8 elements, Supplementary Fig. 3a). Since changes in mechanics, such as stress and strain, primarily occur at locations where the bacterial surface is in contact with the substrate surface, “contact surfaces” were defined by partitioning the lower half of the bacterial surface and the corresponding part of the surface of the substrate into “contact surfaces” (surfaces abcde and fgh, respectively, in Supplementary Fig. 3c); the cellular contact surface is half the entire lower cellular surface and the substrate contact surface is the projection of the cellular contact surface on the substrate. “Contact surfaces” were specifically assigned finer mesh (5 nm mesh size instead of 15 nm in the rest of the model) to allow better resolution of mechanical changes. The contact was formulated as frictionless and ‘hard contact’ along the normal direction.

A typical simulation comprised two steps. In the first step, an “inflating” turgor pressure was applied to the inner surface of an undeformed and free-floating cell envelope. During “inflation,” the cellular mid-plane (curve abc in Supplementary Fig. 2b) was not allowed to move vertically. In the second step, the established turgor pressure was maintained, and the mid-plane was displaced downward by 150 nm to achieve contact between the cell envelope and the substrate. Incremental displacement was applied in ABAQUS and, at every increment at which the bacterium and substrate were in contact, the simulation was analyzed to determine the stresses and strains on bacterial envelopes. Two simulations were performed: one with a stiff substrate and one with a soft substrate (Supplementary Table 1).

We investigated the stress and strain state of four representative shell elements (in the bacterium) that are within the contact surface (inset of Fig. 2b). Stresses were normalized to the respective values at the end of the first step; strains were computed as the net logarithmic strain change between the first and second step. We interpreted the strain state on the innermost section point of a shell element (i.e., on the interior of cell wall) as that experienced by the inner membrane, based on the assumption that the inner membrane is constantly pressed against the cell wall by turgor pressure. The CAREA output variable in ABAQUS was requested at every increment to keep track of the total area in contact as displacement grew. The cell volume enclosed by cell envelopes was calculated using the BOUNDARY syntax in MATLAB (MATLAB 2020 (R2020b), The MathWorks Inc., Natick, MA, USA).

To analyze convergence, the mesh size within the contact surfaces was varied from 30, 25, 20, 15, 10, 7.5, 5, to 2.5 nm (Supplementary Fig. 4j). The circumferential stress on the outer membrane at element #1 (inset of Fig. 2b) was compared at different mesh sizes, and it was found that the mesh size we adopted in our modeling is within the convergence range.

To model surface adhesion by assigning boundary conditions in the form of forces (i.e., the adhesion force scheme), the first step was simply applying turgor pressure to a stress-free cell envelope, the same as the first, “inflation”, step in the displacement scheme described above. In the second step, the mid-plane of the cell envelope was displaced toward the substrate by 90 nm to achieve initial contact and hence easier convergence in the following step. In the last step, vertical, attractive surface tractions with a magnitude of 110 kPa were applied over the contact surfaces of cell envelopes and substrates, resulting in further contact between the two (Supplementary Fig. 3c).

### Levels of intracellular sodium ions in surface-adhered bacteria

In the presence of excess external Na^+^, sodium enters cells through mechanosensitive ion channels that are activated by mechanical tension in the membrane. The assay measuring the intracellular sodium level was done using procedures reported previously^83^ with some modifications. Bacteria were cultured in Tryptone broth (T-broth, 1% tryptone and 0.5% NaCl) at 37 °C with shaking. Two mL of day culture were centrifuged using an Eppendorf Centrifuge 5810 R at 2000 × *g* for 2 min, and the supernatant was discarded. Pelleted bacteria were resuspended in 2 mL potassium chloride (KCl) buffer (10 mM potassium phosphate composed of 5.4 mM K_2_HPO_4_ and 4.6 mM KH_2_PO_4_, 135 mM KCl (Sigma-Aldrich, P9333), pH 7.0) and mixed using a Fisherbrand analog vortex mixer (02215365). The suspension of bacteria in the KCl buffer was then centrifuged (2000 × *g* for 2 min) to pellet bacteria; then, pelleted bacteria were resuspended and mixed in fresh KCl buffer again, as described above. This washing procedure was repeated three times in total. Bacteria were then resuspended and mixed by vortex in 2 mL ethylenediaminetetraacetic acid (EDTA) buffer (the KCl buffer plus 10 mM EDTA (Sigma-Aldrich, EDS)) and left for 10 min at room temperature. The suspension of bacteria in the ETDA buffer was centrifuged at 2000 × *g* for 6 min, and the supernatant was discarded. The resulting pellet of bacteria was resuspended in 2 mL KCl buffer and mixed by vortexing. The resulting bacteria suspension in KCl buffer was centrifuged (2000 × *g* for 5 min), and pelleted bacteria were resuspended and mixed in fresh KCl buffer again. This washing procedure was repeated three times in total. Then, bacteria were resuspended in 750 μL of loading buffer, consisting of 40 μM Sodium Green (sodium ion fluorescence indicator, ThermoFisher Scientific, S6901) in KCl buffer, and left for 30 min in the dark at room temperature. The stock solution of Sodium Green (1 mM) was freshly prepared at each experiment by dissolving Sodium Green in dimethyl sulfoxide (DMSO, Sigma-Aldrich, W387520). The suspension of bacteria in the loading buffer was centrifuged at 2000 × *g* for 5 min, and the supernatant was discarded. The resulting pellet of bacteria was resuspended in 750 μL NaCl buffer (10 mM potassium phosphate composed of 5.4 mM K_2_HPO_4_ and 4.6 mM KH_2_PO_4_, 135 mM NaCl, pH 7.0) and mixed by vortexing. The resulting bacterial suspension in NaCl buffer was centrifuged (2000 × *g* for 5 min), and pelleted bacteria were resuspended and mixed in fresh NaCl buffer again. This washing procedure was repeated three times in total. Bacteria loaded with Sodium Green were resuspended in 400 μL NaCl buffer, and16 μL of this bacterial suspension was added into one imaging spacer (Grace Bio-Labs, SS1X13, 13 mm diameter × 0.12 mm depth) adhered to a coverslip, which was then sealed by an agarose-coverslip composite (either thick or thin). Bacteria were allowed to attach to the gel composite for 1 h.

Then, bacteria attached on thin or thick agarose composites were imaged using an Olympus FV1000 confocal microscope with a 60× oil-immersion objective. The Sodium Green fluorescence indicator was illuminated with a 488 nm laser using standard GFP filter sets and confocal z-stacks were captured by FV10-ASW version 4.2 software. At least 15 stacks were taken at different fields of view for each sample. Each experiment was repeated three times independently. The fluorescence intensity of each bacterium was measured using Fiji. Spinning bacteria were excluded from the analysis. The section below describes the calibration done to account for different attenuations of light by gels of different thicknesses.

### Calibrating for light attenuation by gels of different thicknesses for intracellular sodium ion measurements

Fluorescent polystyrene polymer beads (Bangs Laboratories, Inc., Dragon Green, FSDG004, diameter 1 μm) were used to assess the effect of attenuation of exciting and emitted light passing through gels of different thicknesses. Beads were diluted 1000 times in NaCl buffer (10 mM potassium phosphate composed of 5.4 mM K_2_HPO_4_ and 4.6 mM KH_2_PO_4_, 135 mM NaCl, pH 7.0). 16 μL of this bead suspension was added into one imaging spacer (Grace Bio-Labs, SS1X13, 13 mm diameter × 0.12 mm depth) adhered to a coverslip, which was then sealed by an agarose-coverslip composite (either thick or thin). Beads attached on agarose gel composites were imaged using an Olympus FV1000 confocal microscope with a 60× oil-immersion objective. Fluorescent beads were illuminated with a 488 nm laser using standard GFP filter sets and confocal z-stacks were captured by FV10-ASW version 4.2 software. Supplementary Fig. 6a shows the average fluorescence intensity of beads attached on thick and thin agarose gel composites. Each data point was measured using one photomultiplier tube (PMT) voltage, and by changing the PMT voltage, we obtained an exponential fitted curve to the dataset. Each voltage has 5 different fields of view for one gel sample, and each field of view has about 60-70 beads.

To calibrate the measured intensity of bacterial fluorescence for attenuation by hydrogels, the exponential fitted curve (Supplementary Fig. 6a) was used to convert the fluorescence intensity of each bacterium attached on the thin gel into the equivalent intensity on the thick gel. To do this, the fluorescence intensity of a bacterium was substituted in for the variable x in the fitted equation (Supplementary Fig. 6a), and the y-value, corresponding to intensity on the thick gel, calculated. The resulting converted (or calibrated) fluorescence intensities of bacteria on thin gels were compared with the measured intensities of bacteria on the thick gel.

### Speeds of surface-adhered bacteria during bacterial accumulation

An adhesive imaging chamber (Grace Bio-Labs, PCI-A-2.5, 20 mm diameter × 2.6 mm depth) was filled with 650 μL of bacterial suspension in LB medium and then sealed by a coverslip coated with an agarose gel (either thick or thin). For the next hour, surface-attached bacteria on gel surfaces was observed using an Olympus IX71 inverted phase contrast microscope with a 60× oil-immersion objective. The microscope stage was enclosed within an incubator chamber heated to 37 °C.

Fifteen time-lapse sequences taken at different fields of view for each sample were captured by a Hamamatsu digital camera C11440 controlled by MetaMorph Advanced version 7.7.6.0 software. The acquisition rate was one frame per 0.42 s, and each sequence had 150 frames (total recording time for each sequence was 62.6 s). Each experiment was repeated three times independently.

Trajectories of bacterial centers-of-mass were tracked and the bacteria speed during each interval (0.42 s) was measured using the TrackMate plugin in Fiji^84,85^. The average speed of each tracked bacterium over the tracking period was collected for data analysis. Near-surface swimming bacteria, which displayed a much higher instantaneous speed and a much shorter tracking period (less than 5 s) than surface-attached bacteria, were also tracked by the software. We excluded these swimming bacteria from data analysis.

### Measurement of fractions of surface motility during bacterial accumulation

Among the total tracked surface-attached bacteria, some bacteria remained stationary on surfaces, and the others were motile showing spinning or twitching surface motility. The time-lapse sequences taken to track bacterial trajectories were projected into single-frame images using the ZProjection function (with minimum intensity projections) in Fiji. Projections of stationary bacteria showed the same bacterial cell size as seen in a single time slice-slice. Projections of spinning bacteria reflected motion in a circle, with bacteria appearing as the petals of a “daisy”. Projections of twitching bacteria showed an irregular shape. Using these projections, the number of stationary, spinning and twitching bacteria was counted and recorded. The number of motile bacteria was the sum of the number of spinning bacteria and that of twitching bacteria.

### Measurement of detachment events during bacterial accumulation

The tracks of some surface-attached bacteria had shorter durations than the total recording time (62.6 s). The positions of such short tracks were given by the TrackMate plugin of Fiji. We manually observed bacteria at these locations to determine whether the abbreviated length of the track arose from a bacterium detaching from the surface or from a bacterium newly attaching during the recording period. The number of detachment events was recorded.

### Microscopy measurement of growth of bacteria on surfaces

Bacterial growth curves on surfaces were measured on agarose gel composites. After allowing bacteria suspended in LB medium to accumulate on a gel surface at 37 °C for an hour, surfaces were gently rinsed twice with fresh LB medium and incubated with fresh LB medium at 37 °C. Rinsing and incubation with fresh LB medium was repeated hourly.

After 1, 2, 3, 4, 5, and 6 h of incubation, sample surfaces were gently washed with PBS twice, and then visualized an Olympus IX71 inverted phase contrast microscope with a 60× oil-immersion objective. Images were taken by a QImaging EXi Blue CCD camera controlled by QCapture Pro-6 software and processed using the particle analysis function (for counting single bacteria) and the multi-point function (for counting bacteria in micro-colonies and in clusters) in Fiji, to quantitatively determine the numbers of bacteria on surfaces (i.e., areal density of bacteria (number of bacteria/mm^2^)). Each time point was done for two replicate samples, and at least 12 fields of view were randomly chosen for each replicate. Samples used for measurement at one time point were not used for further incubation or later measurements, i.e., the measurement at each time point was done independently.

### Construction of complemented mutant strains and plasmids

Strains and plasmids used to construct complemented mutant strains and primers used for plasmid construction are listed in Supplementary Table 2. Multicopy plasmids pMQ72 and pMQ70^86^ were used for expression of PilT and PilY1, respectively. Plasmids were designed by homologous recombination using the yeast machinery^86^ or by Gibson assembly^87^ using NEBuilder HiFi DNA Assembly^®^ (NEB, Boston, MA), as previously described. All inserts were sequenced to confirm the integration of the correct sequence. Plasmids were then isolated from *E. coli* S17-λ-pir overnight-grown strains and then electroporated into *P. aeruginosa* PAO1 and grown on the appropriate antibiotic selection plates, as reported previously^86^.

### Microscopy measurement of growth of complemented strains on surfaces

For plasmid maintenance in complemented strains, LB agar and LB liquid medium were supplemented with 60 µg/mL gentamycin for *∆pilT pBAD::pilT* and with 150 µg/mL carbenicillin for *∆pilY1 pBAD::pilY1*. Bacterial growth curves on surfaces were measured on thick and thin agarose gel composites. The LB medium used for rinsing and incubating gel composites with accumulated bacteria was supplemented with 0.025% arabinose for plasmid induction. The overnight culture was first diluted by 1:100 in the LB medium supplemented with arabinose and then grown at 37 °C with shaking until the diluted suspension reached an optical density (OD_600_) of 0.7 for proper plasmid induction before growth curves were measured. With this exception, growth was measured experimentally as also done for the microscopy growth assays described above for *P. aeruginosa* PAO1 WT and the *ΔpilT* and *ΔpilY1* mutants. Each time point was done for one replicate sample, and at least 15 fields of view were randomly chosen for each replicate. Samples used for measurement at one time point were not used for further incubation or later measurements.

### Statistical analysis of exponential growth rate

The exponential regression (f(*t*) = A*e*^*αt*^) for exponential phase datasets was transformed into the linear equation ln(f(*t*)) =ln(A) + *αt*, using Microsoft Excel. Statistical significance of slopes of the linear equation, i.e., bacterial growth rate *α* on gels was determined by ANCOVA testing using the R programming language.

### Measurement of c-di-GMP signaling during bacterial accumulation and growth on surfaces

A suspension of bacterium in LB medium was prepared as described in the section above on assaying bacterial accumulation.

Both the liner cover of the adhesive side and the polycarbonate cover of the non-adhesive side of an imaging chamber (Grace Bio-Labs, PCI-A-2.5, 20 mm diameter × 2.6 mm depth) were peeled off by a tweezer. The adhesive side of the chamber was adhered to a coverslip coated with an agarose gel (either thick or thin). The chamber was then filled with 650 μL of bacterial suspension in LB medium and a coverslip was placed on top of the chamber.

As bacteria accumulated on the gel surface for the next hour, bacteria containing either the reporter plasmid pCdrA::*gfp* or the control plasmid pMH487 were imaged using an Olympus FV1000 confocal microscope with a 60× oil-immersion objective. Bacteria were illuminated with a 488 nm laser using standard GFP filter sets and confocal z-stacks were captured by FV10-ASW version 4.2 software. The microscope stage was enclosed within an incubator chamber heated to 37 °C.

After one hour of bacterial accumulation, the coverslip on top of the imaging chamber was removed, the bacterial suspension in the imaging chamber was gently removed and replaced by 650 μL of fresh LB medium using a pipette, and a new coverslip was placed on top of the imaging chamber. The fresh LB medium was stored at 37 °C before each use. For the following hour of bacterial growth on gel surfaces, bacteria were imaged using confocal microscope as described above. At hourly intervals, the LB medium in the imaging chamber was removed and replaced by fresh LB medium. The experiment was carried on until the end of two hours after the onset of exponential growth indicated in Fig. 5d–f. Each experiment of bacteria containing the reporter plasmid pCdrA::*gfp* was repeated three times independently. Each experiment of bacteria containing the control plasmid pMH487 was repeated twice independently.

The sets of images collected within each 15 min time interval were analyzed separately. Images taken during the first 15 min interval after each replacement of medium were excluded from analysis to avoid including any effects of temperature fluctuation on c-di-GMP signaling. For the second, third, and fourth 15 min interval in each hour, the fluorescent intensity of each bacterium was measured using Fiji, and calibrated using the following bead calibration for light attenuation by gels of different thicknesses. Then the intensity of each bacterium containing the reporter plasmid pCdrA::*gfp* was normalized by subtracting the average per-cell intensity of an ensemble of cells carrying a control plasmid for producing GFP that lacks the *cdrA* promoter (pMH874)^13^.

### Calibrating for light attenuation by gels of different thicknesses for c-di-GMP measurements

We used an approach very similar to the one we developed previously^52^. Fluorescent polystyrene polymer beads (Bangs Laboratories, Inc., Dragon Green, FSDG004, diameter 1 μm) were diluted 1000 times in LB medium. Both the liner cover of the adhesive side and the polycarbonate cover of the non-adhesive side of an imaging chamber (Grace Bio-Labs, PCI-A-2.5, 20 mm diameter × 2.6 mm depth) were peeled off by a tweezer. The adhesive side of the chamber was adhered to a coverslip coated with an agarose gel (either thick or thin). The chamber was then filled with 650 μL of the bead suspension in LB medium and a coverslip was placed on top of the chamber.

Beads attached on agarose gel composites were imaged using confocal microscopy, as described in the bead calibration for measurements of intracellular sodium ions. The microscope stage was enclosed within an incubator chamber heated to 37 °C. Supplementary Fig. 6b shows the average fluorescence intensity of beads attached on thick and thin agarose gel composites. Each data point was measured under one PMT voltage, and by changing the PMT voltage, we obtained an exponential fitted curve to the dataset. Each voltage has 5 different fields of view for one gel sample.

To calibrate the measured fluorescence intensity of GFP for attenuation by hydrogels, the exponential fitted curve (Supplementary Fig. 6b) was used to convert the fluorescence intensity of GFP in each bacterium on the thin gel into the equivalent intensity on the thick gel. To do this, the fluorescence intensity of GFP in a bacterium was substituted in for the variable x in the fitted equation (Supplementary Fig. 6b), and the y-value, corresponding to intensity on the thick gel, calculated. The resulting converted (or calibrated) fluorescence intensities of GFP in bacteria on thin gels were compared with the measured intensities of GFP in bacteria on the thick gel.

### Bacterial accumulation and growth on glass and bulk agarose gel surfaces

Bacterial accumulation and growth on glass and bulk agarose gel surfaces were done with WT and mutants that did not contain any plasmid. A suspension of bacteria in LB medium was prepared as described in Method in the manuscript on assaying bacterial accumulation on thin and thick gel composites.

Glass slides were sonicated in 70% ethanol for 15 min and then dried with nitrogen before use. Both the liner cover of the adhesive side and the polycarbonate cover of the non-adhesive side of an imaging chamber (Grace Bio-Labs, PCI-A-2.5, 20 mm diameter × 2.6 mm depth) were peeled off using tweezers. The adhesive side of the chamber was adhered to a glass slide. The chamber was then filled with 750 μL of bacterial suspension.

3% (w/w) agarose solution was autoclaved, and 500 μL of agarose solution was pipetted into each well of a 24-well plate (15.6 mm diameter of well). The plate was cooled at 4 °C for 2 min, resulting in gelation. Each well was filled with 456 μL of bacterial suspension on top of the bulk gel surface. The height of the bacterial suspension above the glass slide and the bulk gel surface was the same.

To measure the initial accumulation of bacteria after one hour at 37 °C, the bacterial suspension was removed and the glass and bulk gel surfaces were then gently washed with PBS three times. The imaging chamber on the glass surface was removed and the bulk gel was gently removed from the well. Each sample surface was sonicated in 15 mL PBS for 20 min to detach accumulated bacteria. The bacterial concentrations in the 15 mL PBS were measured using a conventional serial dilution method, with the dilutions spread onto the surfaces of agar plates^88^. The agar plates were cultivated at 37 °C for 18 h; the numbers of colonies cultured from the serial dilutions were then counted and the measured counts converted to colony-forming unit (CFU) per mL after multiplication with the dilution factor. Sample surface area was ~314 mm^2^ for glass and ~191 mm^2^ for bulk gel. This was used to determine areal density of bacteria (CFU/mm^2^) on the sample surface.

To measure bacterial growth after the initial one-hour accumulation, the bacterial suspension was removed from the sample surface and replaced by fresh LB medium, which was stored at 37 °C before each use. At hourly intervals thereafter, the numbers of bacteria on surfaces were determined using the plate-counting method described above. Also, at hourly intervals, the LB medium was removed and replaced by fresh LB medium. Each sample has two replicates and each experiment was repeated twice independently.

### Reporting summary

Further information on research design is available in the Nature Research Reporting Summary linked to this article.

### Supplementary information

supplementary material

reporting summary

## Data Availability

The datasets that support the findings of this study are available from the corresponding author on reasonable request.

## Code availability

The code used to support the findings of this study is available from the corresponding author on reasonable request.
