# Supplementary material for: The accumulation and growth of Pseudomonas aeruginosa on surfaces is modulated by surface mechanics via cyclic-di-GMP signaling
Source: NPJ Biofilms Microbiomes. 2023 Oct 10;9:78. doi: 10.1038/s41522-023-00436-x (PMC10564899; doi:10.1038/s41522-023-00436-x)
Supplement: Supplementary file 1 — supplementary material [file 41522_2023_436_MOESM1_ESM.pdf]

Supplementary Information for

The accumulation and growth of *Pseudomonas aeruginosa* on surfaces is modulated by surface mechanics via cyclic-di-GMP signaling

Liyun Wang[1,**], Yu-Chern Wong[1,2,**], Joshua M. Correira[3], Megan Wancura[3], Chris J Geiger[4], Shanice S Webster[4], Ahmed Touhami[5], Benjamin J. Butler[6], George A. O'Toole[4], Richard M. Langford[6], Katherine A. Brown[6,7], Berkin Dortdivanlioglu[8], Lauren Webb[3], Elizabeth Cosgriff-Hernandez[9], Vernita D. Gordon[1,10,11,*]

*Corresponding author: Vernita D. Gordon
Email: gordon@chaos.utexas.edu

This PDF file includes:

Supplementary Discussion
Supplementary Figures 1 to 7
Supplementary Tables 1 to 2
Supplementary References

Supplementary Discussion

Bacterial strains used. All bacterial strains used were *P. aeruginosa* strain PA01, which is a standard laboratory-adapted strain [1]. The wild-type (WT) has functional pili and a flagellum, and makes the cell-surface-associated protein PilY1. The Δ*pilA* mutant does not have pili, and the Δ*fliC* mutant does not have a flagellum. The Δ*pilT* mutant does not have a functional pilus retraction motor, and therefore is immotile on surfaces. The Δ*pilY1* mutant does not make the cell-surface-associated protein PilY1. All of the strains used in this study have functional *cdrA* genes. The plasmid P_{CdrA}::*gfp* we used in this study, is a verified reporter for c-di-GMP, which is a transcriptional fusion between the cyclic di-GMP-responsive *cdrA* promoter and a gene encoding GFP. The c-di-GMP production, which was described by the transcription level of *cdrA* gene, could be seen in Figure 5 in the main text. PilY1 has previously been implicated in cyclic-di-GMP production. PilT has been implicated in cyclic-AMP signaling.

Bacterial accumulation on glass and bulk agarose gel surfaces. Initially, we chose glass and bulk agarose gel as test surfaces of highly contrasting stiffness (glass, 82 GPa; 3% w/w agarose gel, 45 kPa). When we incubated WT, Δ*fliC*, Δ*pilA*, and Δ*pilY1* for one hour with those two surfaces, we found that an order of magnitude more WT accumulated on rigid glass than on soft agarose (Supplementary Fig. 2a). Δ*fliC* and Δ*pilA* accrued to both surfaces in much smaller numbers, but the ratio of accumulated bacteria on glass and agarose was not significantly different from that for WT (P = 0.36 for WT vs. Δ*fliC*; P = 0.63 for WT vs. Δ*pilA*, χ^2 test). However, the same ratio for Δ*pilY1* was significantly smaller than that for WT (P <0.001, χ^2 test), reflecting Δ*pilY1* accumulating more equally on the two surfaces (Supplementary Fig. 2a). This suggests PilY1 as a candidate mechanosenory element that may allow *P. aeruginosa* to sense surface stiffness during bacterial accumulation. Due to the very highly defective attachment of Δ*fliC* on both glass and agarose surfaces and its high ratio of accumulated bacteria on glass and agarose (i.e., flagellar filament did not contribute much to bacterial mechanosensing of surface stiffness), Δ*fliC* cells were not used in subsequent experiments.

The choice of substrate materials to make surfaces with different stiffness. For more careful subsequent investigation, to avoid the effect of different surface chemical properties, we decided to fabricate surfaces of different stiffness using the same material. For instance, by varying the mass ratio of base to curing agent, the stiffness of poly(dimethylsiloxane) (PDMS) can be varied. Some researchers have found that bacteria accumulated more on soft PDMS than on stiff PDMS surfaces [2]; however, polymer beads are also more likely to attach passively to soft PDMS than to stiff PDMS [3], which suggests that soft and stiff PDMS surfaces prepared by varying the ratio of base to cure also have different adhesive properties from each other. This presents the possibility that that PDMS surface adhesivity rather than stiffness may be governing attachment [3]. In contrast, another group used the same mass ratio of PDMS base to curing agent but changed the thickness of PDMS layer spin-coated on coverslips, has reported that more bacteria accumulated on thinner (i.e. stiffer) PDMS surfaces [4]. Similarly, Kolewe et al. found that more bacteria accumulated on poly(ethylene glycol) hydrogels when gels are stiffer [5]. Therefore, in the state of the art, it is still contradictory how bacteria actively respond to the stiffness of the surface to which they attach. To eliminate effects arising from physicochemical properties of surfaces other than stiffness (such as differences in adhesivity), we fabricated thin and thick hydrogels with the same chemical compositions coated on glass coverslips (Fig. 1a) and used gel thickness to manipulate the effective stiffness of a gel-coverslip composite (Fig. 1d).

Using a different fabrication method for thin gel than for thicker gels could raise concerns about whether different fabrication methods generated unwanted alterations of gel properties other than thickness. To address this, we examined the chemical composition and surface topographies of thin and thick gels, as follows:

The chemical properties of hydrogel-coverslip composites with different thickness. Our infrared spectroscopy measurements suggest that varying hydrogel thickness does not alter the chemical composition of hydrogel samples as samples with different thickness exhibit similar spectra (Fig. 1b and Supplementary Fig. 1b). For thin agarose hydrogel, the peaks are observed at 1155 (–C–O–C groups), 1072 (glycosidic linkage), 933, 889, and 775 cm^{-1} (3,6-anydro-β-galactose skeletal bending). Similarly, for

thick agarose hydrogel, the peaks are observed at 1153, 1072, 933, 889, and 773 cm^{-1} (Fig. 1b). For thin alginate hydrogel, the peaks are observed at 1597, 1419 (symmetric and asymmetric stretching vibrations of –COO–, respectively), and 1034 cm^{-1} (C–O stretching vibration absorption of uronic acid in the G chains and M chains); similarly for thick alginate hydrogel, the peaks are observed at 1595, 1417, and 1034 cm^{-1} (Supplementary Fig. 1b). The infrared spectroscopy peaks we identify in agarose [6] and alginate [7] hydrogels are consistent with the literature.

Surface topography on thick and thin hydrogel composites. Cryo-electron microscopy imaging showed no discernable difference between the surface topographies of thick and thin composite substrates (Supplementary Fig. 1d and e).

Effective stiffness of hydrogel-coverslip composites – analytical modeling. When a composite substrate consisting of soft gel on top of rigid glass is subjected to a uniform external loading normal to the gel surface, *N*, linear elasticity yields the following relations for the normal strains, ε as:

$$\varepsilon_{\text{gel}} = \frac{N}{E_{\text{gel}}} \quad [1]$$

$$\varepsilon_{\text{glass}} = \frac{N}{E_{\text{glass}}} \quad [2]$$

, where E_{gel} and E_{glass} are the Young's modulus of the gel and the glass, respectively, and we assume isotropy in both materials. Upon loading, the deformed thickness, t' in the gel and the glass can be expressed in terms of the undeformed thickness, t, and the normal strains:

$$t'_{\text{gel}} = t_{\text{gel}}(1 + \varepsilon_{\text{gel}}) \quad [3]$$

$$t'_{\text{glass}} = t_{\text{glass}}(1 + \varepsilon_{\text{glass}}) \quad [4]$$

The total normal strain of the gel-glass composite is, by definition:

$$\varepsilon_{\text{effective}} = \frac{(t'_{\text{gel}} + t'_{\text{glass}}) - (t_{\text{gel}} + t_{\text{glass}})}{t_{\text{gel}} + t_{\text{glass}}} \quad [5]$$

Recognizing the relation such that $N = E_{\text{effective}} \cdot \varepsilon_{\text{effective}}$ and substituting Supplementary Equation 1-5 into this relation, we have:

$$E_{\text{effective}} = \frac{N}{\varepsilon_{\text{effective}}} = \frac{t_{\text{gel}} + t_{\text{glass}}}{t_{\text{gel}}/E_{\text{gel}} + t_{\text{glass}}/E_{\text{glass}}} \quad [6]$$

Given that t_{gel} and t_{glass} are similar in magnitude and E_{glass} is several orders of magnitude greater than E_{gel} (~GPa vs. ~kPa, respectively), Supplementary Equation 6 reduces to:

$$E_{\text{effective}} \cong \frac{t_{\text{gel}} + t_{\text{glass}}}{t_{\text{gel}}} E_{\text{gel}} \quad [7]$$

By applying shear loading on the surface of the gel and following derivations like those above, one can also show that the effective shear modulus for the composite, $G_{\text{effective}}$ takes on the same expression as $E_{\text{effective}}$ in Supplementary Equation 7 (or simply recognizing the linear elasticity relation such that $E = 2(1 + \nu)G$ for isotropic materials, where ν is the Poisson's ratio).

The derivations for the composite effective stiffness rely on the assumptions that (1) gel and glass are perfectly bonded; (2) the forces exerted by bacteria on gel surface are sufficiently distributed; and (3) substrate materials are homogeneous. Any violations or compromises of these assumptions would lead to smaller effective stiffness than that predicted by Supplementary Equation 7. Although assumption (3) seems to be valid given that gel is made of randomly oriented polymer chains with a very narrow distribution of pore sizes [8], assumptions (1) and (2) may not hold true. Gel can sometimes be washed away during rinsing; *P. aeruginosa* can pull on surfaces using their type IV pili which have an attachment radius as small as ~1 nm [9] such that the bacteria may exert pulling forces on single polymer chains (~10^0-10^1 nm [10]) rather than on the polymer network. These facts contradict assumptions (1) and (2), respectively. Therefore, we directly probed the surface mechanics of gel-glass composites using nanoindentation as described below.

Effective stiffness of hydrogel-coverslip composites – nanoindentation measurements. The thickness of the thin gel substrate is comparable to the size of bacterial cells, and thus we expect that the cells should be able to sense the presence of the rigid coverslip beneath the gel, resulting in an effective substrate stiffness significantly greater than that of bulk gel. To estimate the effective stiffness of the gel and coverslip composites, we cannot use the Hertzian contact theory, which is the standard theory to fit indentation data and find the Young's modulus of a sample, because its derivation assumes that the substrate is an infinite half-space; this assumption is invalid when substrate thickness is comparable to indentation depth and indenter size [11], which is the case for cells (~10^0 μm) adhering to thin gel (also ~10^0 μm) in our experiments. Therefore, we empirically investigated the effective stiffness of composite substrates with different gel thickness by imposing indentation depths of several microns and examining the resulting indenting load. These measurements show that, to achieve the same indentation depth, a greater load had to be applied to composites with thin gel than to composites with thick gel (Fig. 1e). This indicates that, on bacterial length scales, the thin gel composite has greater effective stiffness than does the thick gel composite.

We validated our nanoindentation measurements by performing standard Young's modulus measurements on thick and thin hydrogel samples, where a very small indentation was imposed (<0.9 μm, which is small compared to the thickness of thin, ~5 μm and that of thick, ~150 μm) and the Hertz contact theory holds true. At small indentation, the composite effect diminishes, and we expect the Young's modulus of thick and thin hydrogel to be the same. Indeed, we see no difference in gel's Young's modulus with different gel thickness and those moduli are consistent with the reported value [5].

Approximating bacterial surface adhesion using displacement scheme in finite element models. Bacterial surface adhesion is a highly complicated process. The initial adhesion is governed by the balance between Lifshitz-Van der Waals, electrostatic, and acid-base interactions between bacteria and surfaces. Following the equilibrium of these interaction forces, physicochemical bond-strengthening occurs, which includes gradual removal of interfacial water, conformational changes of bacterial surface proteins, more bacterial appendages tethering to surfaces, etc., and leads to irreversible adhesion. Meanwhile, bacterial envelopes (and sometimes surfaces as well) deform due to these interactions. The changes in contact area and the distance between bacterial envelopes and surfaces resulted from the deformation further increase adhesive strength, presenting another mechanism for bond-strengthening. After sensing surface adhesion, bacteria initiate extracellular polymeric substances (EPS) production for subsequent biofilm formation and EPS coating on bacterial surface also alters adhesivity between bacteria and surfaces [12]. In short, adhesive strength is heterogeneously distributed over bacterial surface and develops as time grows. The finite element models which used the adhesion force scheme (Supplementary Fig. 3c and d) may oversimplify adhesion by assuming fixed attraction forces uniformly distributed over an arbitrarily prescribed bacterial surface area. The resulting stress state on bacterial envelopes using these models can reflect the physiological stress pattern but its magnitude is greatly dependent on the choice of adhesive properties, which remain ill defined. Therefore, we developed the displacement scheme (Fig. 2b) and characterized the bacterial stress state over a wide range of possible degree of surface adhesion (by assigning varying amount of displacement on bacteria toward surfaces) without explicitly prescribing a set of adhesive properties.

We verified that the displacement scheme generates similar stresses and spatial distributions of stress by comparing these results with those from the adhesion force scheme. It is observed that within the tested displacement range, the displacement scheme generated similar stress magnitudes and patterns as the adhesion force scheme (Supplementary Fig. 3d).

Spatial distribution of bacterial envelope stress/strain upon surface adhesion. The mechanical stresses and contact pressure on the outer membrane exhibited uniform distribution (Fig. 2d and Supplementary Fig. 4a-f) within the central contact zone (element #1-4, inset of Fig. 2b) and were isotropic. However, the stresses in the outer membrane were different near the edge of the contact zone and in the bacterial cap region of the contact zone (Fig. 2b). The reason that the outer membrane can endure nonuniform stress distribution over the whole cell is probably due to some level of solid-like features of the bacterial outer membrane and consequently its structural integrity (lipopolysaccharides and outer membrane proteins have restricted lateral mobility on the outer membrane) [13].

The inner membrane strains were uniformly distributed within the central contact zone (element #1 and 2, inset of Fig. 2b) but anisotropic along the two principal axes: circumferential strain increased and

then plateaued while axial strain monotonically decreased as indentation grew (Supplementary Fig. 4g and h). The strains were also nonuniformly distributed over the whole cell (Supplementary Fig. 4i).

In our bacterial surface accumulation experiments, we observed that the mechanosensitive ion channels on the bacterial inner membrane were activated upon surface adhesion (Fig. 2a), indicating an increase in mechanical strains on the membrane. Nevertheless, our finite element models suggest that the increase in membrane strains upon surface adhesion did not exceed 1% (Supplementary Fig. 4g and h), which is smaller than the threshold strain required to activate mechanosensitive ion channels [14-16]. Similarly, subthreshold membrane strains were found even if we expressed the strains in other in-plane coordinates (a process called coordinate transformation: the magnitude of stress/strain is dependent on the choice of coordinate systems). We first speculated whether holding turgor pressure constant in our models led to underestimation of membrane tension. In the modeling, we observed a decrease in cellular volume as bacteria adhere to surfaces. With the presence of semi-permeable membranes, the volume drop is explained by the efflux of water, leading to a higher concentration of intracellular ions and to the consequential increase in turgor pressure and membrane tension. However, as we further studied the changes in cellular volume, we found it decreased by at most 2% upon surface adhesion (Supplementary Fig. 3b). Thus, simplifying turgor pressure to a fixed value in our models is insufficient to explain the apparent discrepancy between modeling and experiments.

A different possible explanation for this discrepancy lies in the way we computed inner membrane strains. We inferred the mechanical strain state on the inner membrane by equating the strains on the inner surface of the cell wall and those on the membrane. This implicitly assumes that the inner membrane is firmly pressed against the cell wall by the turgor pressure. However, realistically the inner membrane may recede from the cell wall (e.g., the membrane wrinkles, plasmolysis occurs, etc.), resulting in a different strain state between the membrane and the cell wall. Furthermore, informed by the fact that the inner membrane is fluid-like [13], a theoretical study indicated that membrane strains are expected to redistribute and become isotropic and uniform to achieve minimum total potential energy at equilibrium [17]. The strains on the cell wall were clearly anisotropic (Supplementary Fig. 4g and h) and nonuniform (Supplementary Fig. 4i). Equating the strains on the cell wall to those on the inner membrane may not fully capture the fluidic features of the membrane. As a result, to accurately characterize the strain state on the inner membrane, a more sophisticated model, perhaps with the inclusion of another layer of fluidic membrane beneath the cell wall, is needed in the future.

TFP impact the exponential growth dynamics of biofilms. Unlike Δ*pilY1*, the Δ*pilT* mutant (which is deficient in retraction of TFP) had initial c-di-GMP increases on thin and thick gels and subsequent lag phases similar to those of WT (Fig. 5a and c). From this, we conclude that functional TFP are not required for controlling *P. aeruginosa*'s initial c-di-GMP response to surface stiffness during initial accumulation or the consequent lag time in early biofilm growth.

For bacteria suspended in liquid medium, growth rates of planktonic WT, Δ*pilT* and Δ*pilY1* were not significantly different (data not shown). However, on substrates, in the exponential growth phase following the biofilm lag phase, both WT and the Δ*pilY1* mutant had significantly higher growth rates on soft substrates than on stiff. In contrast, the Δ*pilT* mutant had the same growth rate on both soft and stiff composites (Fig. 5d-f). The difference lay in the growth response to stiff substrates: On soft substrates, WT, Δ*pilT,* and Δ*pilY1* all had statistically-similar exponential growth rates ($P > 0.1$ for Δ*pilT* vs. WT and $P > 0.1$ for Δ*pilY1* vs. WT, ANCOVA test). However, on stiff substrates, Δ*pilT* had a significantly higher growth rate than WT ($P < 0.05$, ANCOVA test), although Δ*pilY1* did not ($P > 0.1$, ANCOVA test). This finding implies an important role of functional TFP - specifically the pilus retraction motor PilT - in responding to surface stiffness by modulating the exponential growth phase of biofilms. When PilT was complemented back on an arabinose-inducible plasmid, this restored different growth rates on stiff and soft substrates (Supplementary Discussion and Supplementary Fig. 6d).

On stiff substrates, twitching-capable WT were uniformly distributed on surfaces, without cluster formation, and the Δ*pilT* mutants, which do not twitch, were in tight multilayered clusters (Supplementary Fig. 7a and b). These are in agreement with previous findings for *P. aeruginosa* on rigid glass surfaces, on which twitching allows bacteria to explore space in the plane of the surface [18,19]. PilY1 assists in the twitching motility of *P. aeruginosa* [20,21], and we found that the Δ*pilY1* mutant, which has defective twitching motility, were in loose clusters when growing on thin gels (Supplementary Fig. 7c).

In contrast, on soft substrates, WT, Δ*pilT*, and Δ*pilY1* all formed densely-packed micro-colonies, with no obvious differences between strains (Supplementary Fig. 7d-f). This is consistent with simulations

showing that pilus-driven deformations of very soft surfaces may not generate sufficient force to drive twitching motility [22]. We speculate that *P. aeruginosa* micro-colony expansion on soft hydrogel surfaces may be independent of twitching motility – instead it likely depends on sliding motility, a passive motility mode powered by cell growth [23]. Such an interpretation is consistent with our finding that the exponential growth dynamics of biofilm on soft substrates do not depend on TFP. Together, these findings suggest that the retraction motor PilT and twitching motility might be important for bacterial recognition of stiff surfaces during the exponential growth of biofilms.

Growth dynamics of the complemented strains. The Δ*pilY1/*P_{BAD}*::pilY1* complement populations had a lag time for 2h on thin gels but again, they immediately entered the exponential phase once the incubation began on thick gels (Supplementary Fig. 6c). The Δ*pilT/*P_{BAD}*::pilT* complement populations remained in the lag phase for the duration of the experiments on thin gels whereas they immediately entered the exponential phase once the incubation began on thick gels (Supplementary Fig. 6d). Both the complemented strains exhibited different growth dynamics from the deletion mutants and restored the growth dynamics of WT (Fig. 5d-f), suggesting that PilT and PilY1 were responsible for the differences in growth dynamics between the deletion strains and WT and are the mediators of early biofilm growth as concluded in the main manuscript.

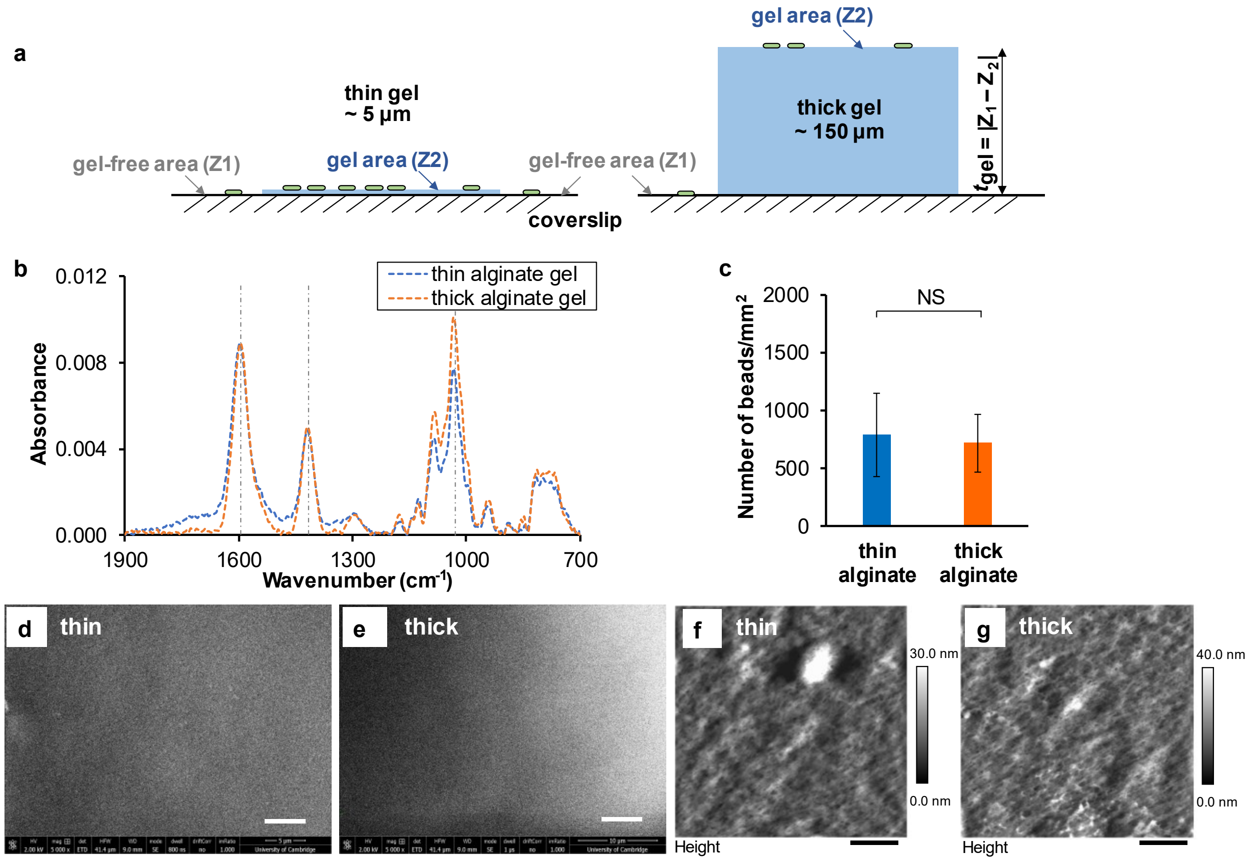

Supplementary Fig. 1. (a) Schematic of setup used to measure gel thickness by microscopy. **(b)** FTIR spectra of alginate gel composites with two thicknesses. One spectrum from each sample is shown here. The dash-dot lines indicate the location of characteristic peaks. N=3. **(c)** The number of beads attached alginate gel composites after incubation with bead suspension in NaCl buffer for 1 h. NS, not significant (*P* = 0.62); ANOVA test. NS indicates that the attachment of beads on thin and on thick gels are not significantly different for alginate gel composites. Data are means ± SD. N=2. **(d and e)** Representative images showing the surface of **(d)** 'thin' and **(e)** 'thick' hydrogels as visualized by Cryo Surface Electron Microscopy. This imaging technique shows no detectable differences between the topographies of thick and thin composites. Scale bar: 5 µm. **(f and g)** AFM images showing the surface topography of **(*f*)** thin and **(g)** thick alginate hydrogels. Scale bar in **(f)**: 400 nm. Scale bar in **(g)**: 600 nm. Greyscale map of height is indicated to the right of each panel.

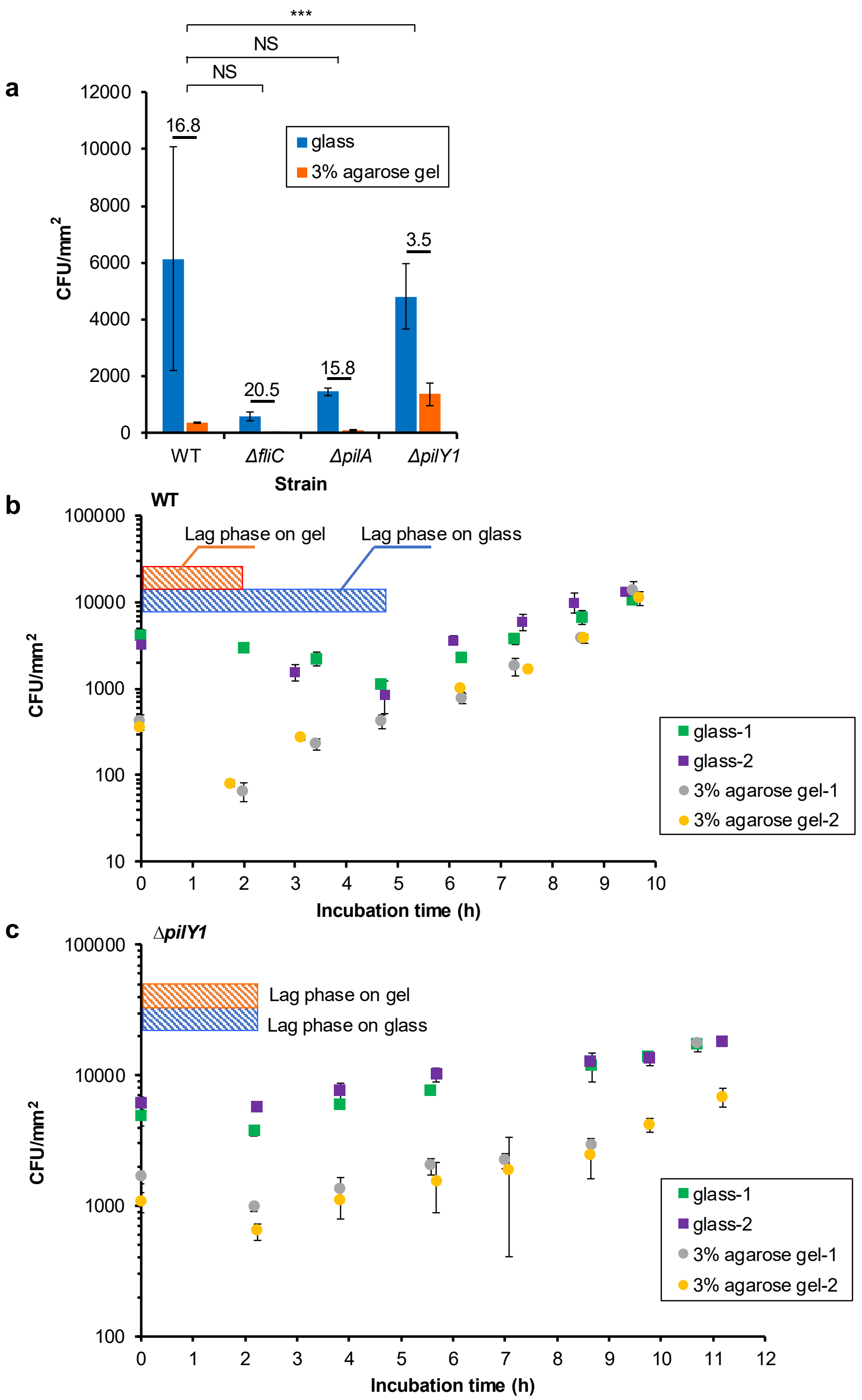

Supplementary Fig. 2. (a) One hour after introducing bacterial suspension to surfaces, the numbers of WT and the Δ*fliC*, Δ*pilA,* and Δ*pilY1* mutants on glass surfaces are respectively 16.8, 20.5, 15.8 and 3.5 times higher than those on bulk agarose gel surfaces. Data are means ± SD. N=4 replicates. NS, not significant (P = 0.36 for WT vs. Δ*fliC*; P = 0.63 for WT vs. Δ*pilA*; χ^2 test). NS indicates that the ratio of accumulated mutant bacteria on glass and agarose is not significantly different from that for WT. ***P <0.001; χ^2 test. *** indicates the ratio of accumulated the Δ*pilY1* mutant on glass and agarose is significantly smaller than that for WT. Growth curves of WT **(b)** and the Δ*pilY1* mutant **(c)** on glass and bulk agarose gel surfaces,

determined by plate counting method. The first timepoint shown, time = 0 h, occurs after bacteria have been allowed to accumulate to surfaces for one hour. Replicate experiments are indicated by -1 and -2 on the same surface type. Color blocks show the average length of the lag phase on glass (blue) and bulk gel (orange) surfaces. Data are means ± SD. N=4 (2 biological replicates for each case; each biological replicate was done with two technical replicates. These data were taken by suspending the bacteria on the surface in liquid medium, serial dilution, plating onto nutrient agar, and counting the number of colonies that grew. The number of colony-forming-units (CFUs) per unit area of substrate is used as a measure of the bacterial population on the substrate.

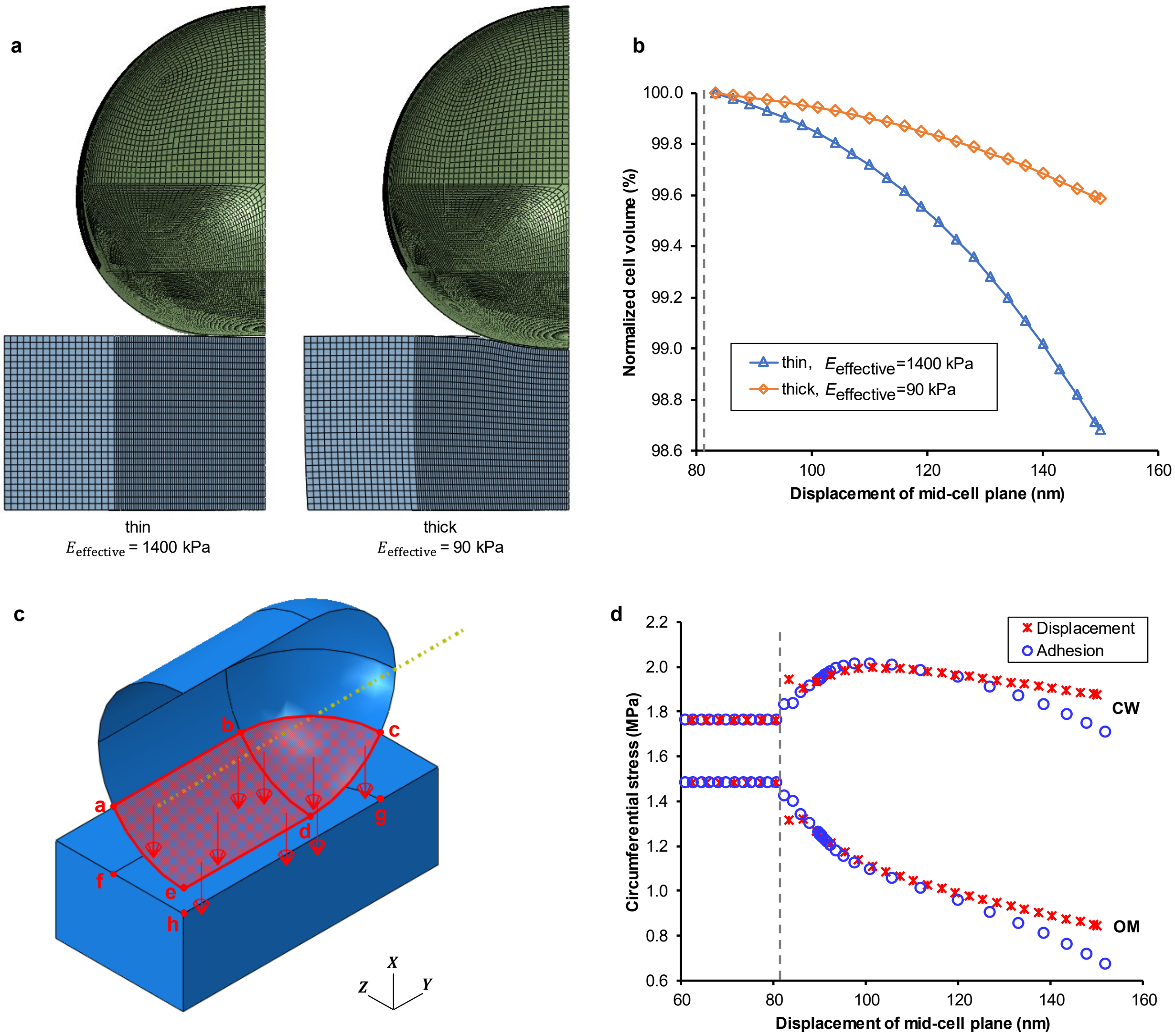

Supplementary Fig. 3. The configuration change **(a)** and cell volume change **(b)** as a cell envelope attaches to thin and thick gel surfaces using finite element models. The mesh lines are rendered and the configurations at a displacement of 150 nm are shown. Cell volume is normalized to that in the free-floating state. **(c)** Approximating bacterial surface adhesion by displacing a cell envelope toward gel sufaces. The schematic illustration of the adhesion force scheme for modeling surface adhesion. Arrows denote the direction of adhesion forces, and the highlighted area denotes the area over which the forces are applied. Note that the same forces with the opposite direction are applied on the gel surfaces but they are not shown here for brevity. **(d)** Comparing the displacement and adhesion force schemes. The circumferential stresses at element #1 with different loading schemes are compared. The dash line denotes when the cell first contacts the substrate. CW: cell wall; OM: outer membrane.

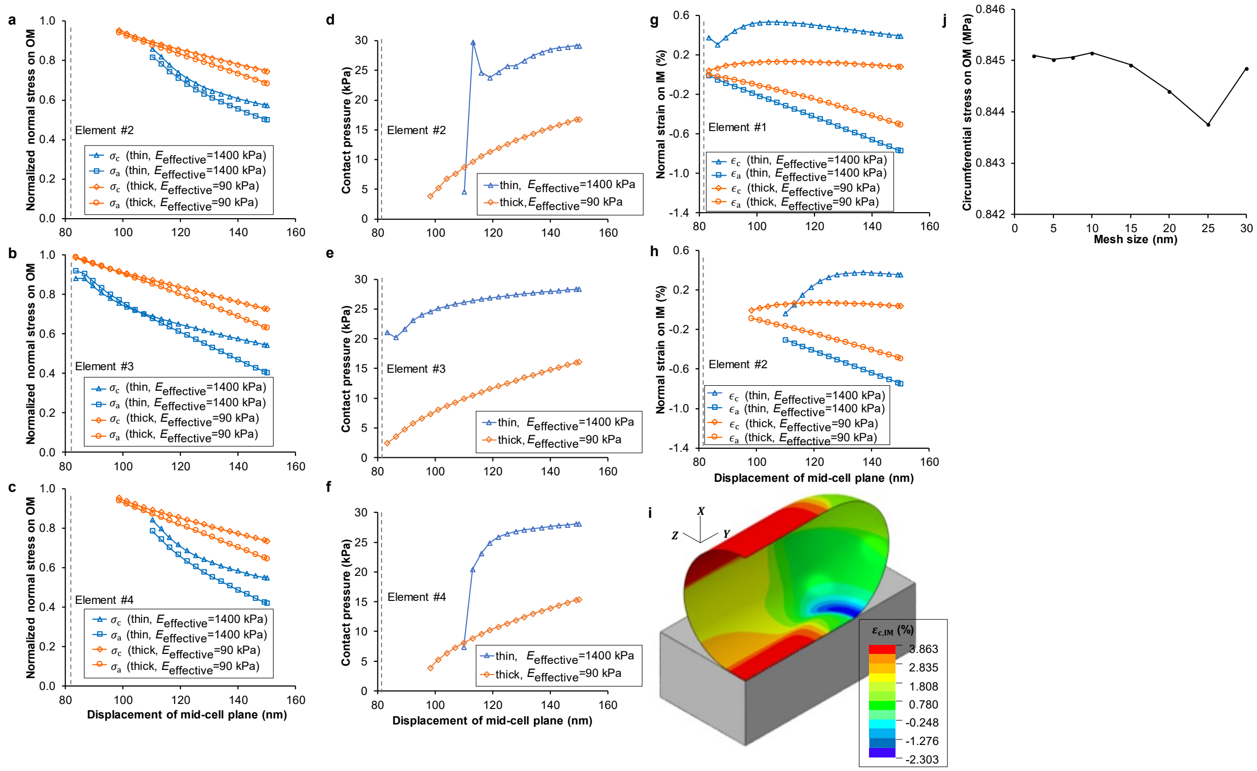

Supplementary Fig. 4. The spatial distribution of outer membrane stresses **(a-c)** and contact pressure **(d-f)**. Stresses are normalized to their respective values during the free-floating state. The dash line denotes when the cell first contacts the substrate. **(g and h)** Mechanical strain state on the inner membrane, and its spatial distribution **(i)**. The strain distribution is illustrated with a model where the substrate is stiff and the displacement is 150 nm. Note that the strain here is calculated based on a stress-free reference state. The mesh lines are hidden and the strains in the substrate are not shown. **(j)** Convergence studies of the finite element models. The circumferential stress of the outer membrane at element #1 is compared at different mesh sizes. In all images, subscript c denotes the circumferential direction and subscript a denotes the axial direction.

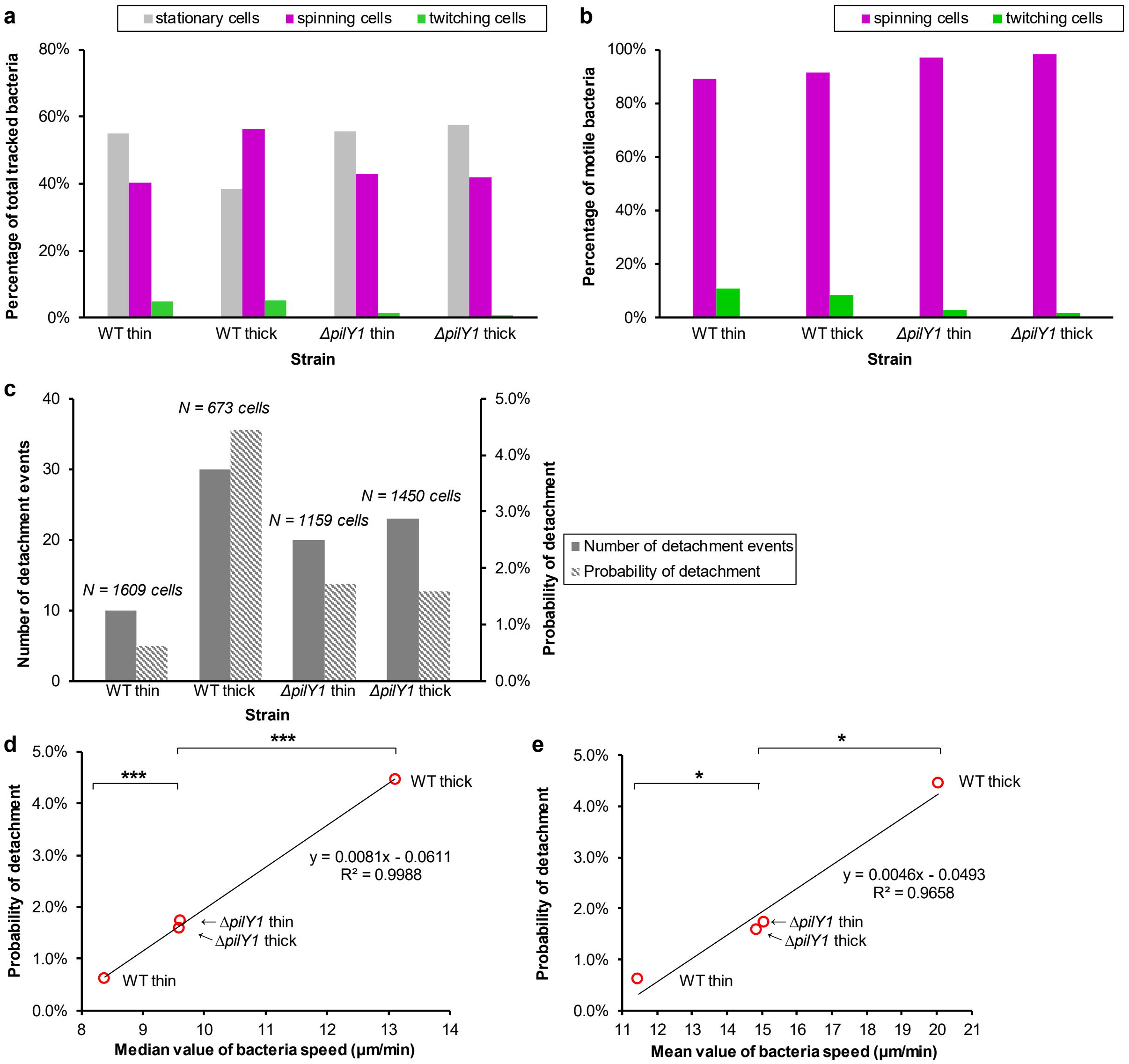

Supplementary Fig. 5. Bacterial surface motility during the first one hour of the accumulation process. **(a)** Among all tracked bacteria on thin or thick agarose gel composites, the percentage of WT and the Δ*pilY1* mutant remaining stationary and showing flagellum-driven spinning motility or TFP-driven twitching motility. **(b)** The percentage of spinning or twitching WT and the Δ*pilY1* mutant, accounting for all motile bacteria attached to surfaces. **(c)** Detachment of adhering WT and the Δ*pilY1* mutant from thin and thick agarose gel surfaces during the first one hour of the accumulation process. WT are significantly more likely to detach from thick gels (30 detachment events among 673 tracked cells) than from thin gels (10 detachment events among 1609 tracked cells) (P <0.001, χ^2 test), while Δ*pilY1* are equally likely to detach from thin and thick gels (P = 0.78, χ^2 test). **(d)** The linear correlation for the median value of bacteria speed. **(e)** The linear correlation for the mean value of bacteria speed. *** P <0.001; Mann-Whitney u test. * P <0.05; based on the 95% confidence interval of mean value of bacteria speed reported in the Main text. These indicate that bacteria speed of the Δ*pilY1* mutant on gels is significantly higher than that of WT on thin gels, but significantly lower than that of WT on thick gels, for both median value and mean value of bacteria speed. Biological replicates N=3 in all cases, with each biological replicate represented by 15 video sequences at randomly-chosen fields of view.

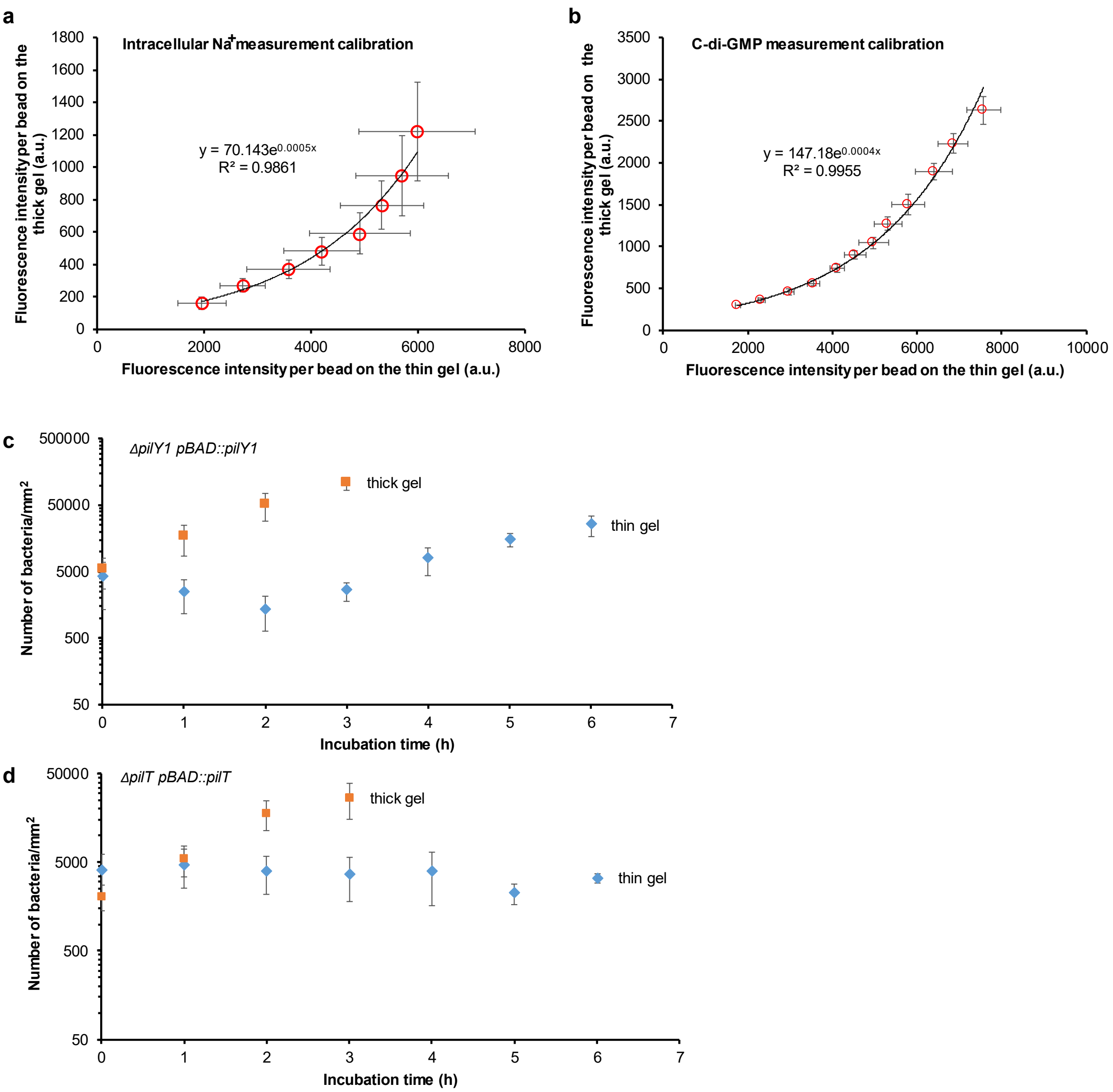

Supplementary Fig. 6. (a and b) The corresponding relations between the mean fluorescence intensity of beads attached on thick agarose gels and on thin agarose gels. **(a)** The relation for intracellular Na^+ measurement, in which beads were imaged within NaCl buffer in an imaging spacer (0.12 mm depth). **(b)** The relation for c-di-GMP measurement, in which beads were imaged within LB medium in an imaging chamber (2.6 mm depth). The exponential equation is the fitted curve to the dataset. Growth dynamics of attached Δ*pilY1*/P_{BAD}::*pilY1*. **(c)** and Δ*pilT*/P_{BAD}::*pilT* complements **(d)** on thin and thick agarose gel composites. Data are means ± SD. The data at 0 time point corresponds to the end of one hour of bacterial accumulation on gel surfaces.

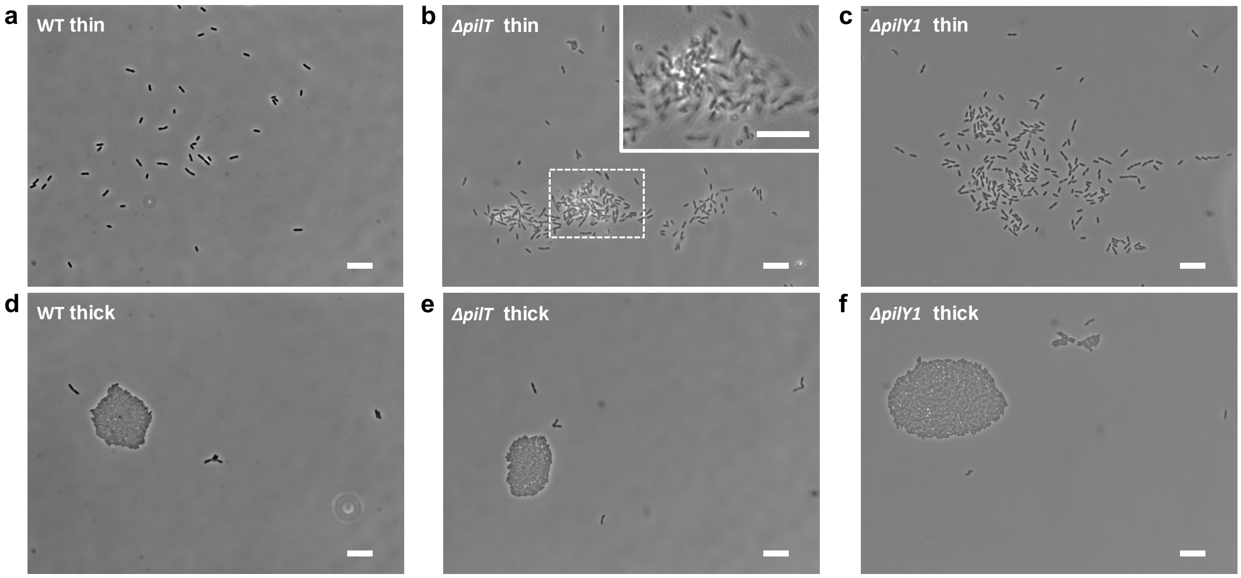

Supplementary Fig. 7. Phase contrast images of WT **(a and d)** and the Δ*pilT* **(b and e)** and Δ*pilY1* **(c and f)** mutants after six hours of incubation on thin and thick agarose gels. The inset in **(b)** shows a magnified image of the upper layer of cells in a micro-colony cluster of Δ*pilT*, in the area indicated by the dotted box. Scale bar: 10 μm.

Supplementary Table 1. Physical/mechanical properties in the finite element models.

Part		Material Model	Parameter[1]		Reference
Outer Membrane (OM)		Neo-Hookean	C_{10}	4.83 MPa	24
			D_1	6 kPa	
Cell Wall (CW)	Trunk	Orthotropic elastic	E_c	45 MPa	25
			E_a	25 MPa	25
			G_{ca}	17 MPa	-
			ν_{ca}	0.34	26
	Cap	Isotropic elastic	E	35 MPa	25
			ν	0.3	-
Whole Cell		-	P_t	30 kPa	27
			t_{OM}	4 nm	28
			t_{CW}	4 nm	
			L	2024 nm	-
			R	414 nm	29
Gel		Isotropic elastic	$E_{gel,bulk}$	44.8 kPa	30
			$E_{gel,stiff}$	1400 kPa	Calculated from Supplementary Equation 7 [2]
			$E_{gel,soft}$	90 kPa	
			ν_{gel}	0.45	-

[1] C_{10}, D_1: neo-Hookean parameters, E: Young's modulus, G: shear modulus, ν: Poisson's ratio
P_t: turgor pressure, t: thickness, L: cell length, R: cell radius
Subscript *c* denotes circumferential direction, and subscript *a* denotes axial direction.

[2] The Young's modulus of bulk agarose (3%) gel reported in Kolewe *et al*'s work [5] was 44.8 kPa. The calculated effective composite moduli of thin and thick agarose gel and glass composites are 1388.8 and 89.6 kPa, respectively. The Young's modulus of bulk alginate (2%, 50 mM $CaCl_2$) gel reported in Nunamaker *et al*'s work [31] was 32.0 kPa. The calculated effective composite moduli of thin and thick alginate gel and glass are 992.0 and 64.0 kPa, respectively.

Supplementary Table 2. Strains, plasmids and primers used in this study.

Strain Name	Genotype/Description	Source
SMC9459	Δ*pilT*/P$_{BAD}$::*pilT* (Gm^r)	This study
SMC9256	Δ*pilY1*/P$_{BAD}$::*pilY1* (Cb^r)	This study

Plasmid	Description	
pMQ70	Expression vector using arabinose inducible expression; Cb^r	(14)
pMQ72	Expression vector using arabinose inducible expression; Gm^r	(14)
pMQ70- P$_{BAD}$::*pilY1*	Plasmid to express the PilY1 protein; Cb^r	(15)
pMQ72- P$_{BAD}$::*pilT*	Plasmid to express the PilT protein; Gm^r	This study

Primer Name	Primer Sequence (5’-3’)	Purpose
pMQ72_pilT_5’	GGGTACCgaaggagatatacatATGGATATTACCGAGC TGCTCGCC	Cloning *pilT* into pMQ72
pMQ72_pilT_3’	GCGAAGCTTtcaGAAGTTTTCCGGGATCTTCgccttct cg	Cloning *pilT* into pMQ72

Supplementary References

1 Holloway, B. W. Genetic Recombination in Pseudomonas aeruginosa. *Microbiology* **13**, 572-581, doi:https://doi.org/10.1099/00221287-13-3-572 (1955).

2 Song, F. *et al.* How bacteria respond to material stiffness during attachment: a role of *Escherichia coli* flagellar motility. *ACS Appl. Mater. Interfaces* **9**, 22176-22184 (2017).

3 Straub, H. *et al.* Bacterial Adhesion on Soft Materials: Passive Physicochemical Interactions or Active Bacterial Mechanosensing? *Adv. Healthc. Mater.* **8**, 1801323 (2019).

4 Peng, Q. *et al.* Three-dimensional bacterial motions near a surface investigated by digital holographic microscopy: effect of surface stiffness. *Langmuir* **35**, 12257-12263 (2019).

5 Kolewe, K. W., Peyton, S. R. & Schiffman, J. D. Fewer bacteria adhere to softer hydrogels. *ACS Appl. Mater. Interfaces* **7**, 19562-19569 (2015).

6 Wang, S. *et al.* Strength enhanced hydrogels constructed from agarose in alkali/urea aqueous solution and their application. *Chem. Eng. J.* **331**, 177-184 (2018).

7 Hu, Y. *et al.* Construction and evaluation of the hydroxypropyl methyl cellulose-sodium alginate composite hydrogel system for sustained drug release. *J. Polym. Res.* **25**, 1-12 (2018).

8 Jiang, L. & Granick, S. Real-space, in situ maps of hydrogel pores. *ACS Nano* **11**, 204-212 (2017).

9 Koch, M. D., Black, M. E., Han, E., Shaevitz, J. W. & Gitai, Z. *Pseudomonas aeruginosa* distinguishes surfaces by stiffness using retraction of type IV pili. *Proc. Natl. Acad. Sci. U.S.A.* **119**, e2119434119 (2022).

10 Lei, J., Li, Z., Xu, S. & Liu, Z. Recent advances of hydrogel network models for studies on mechanical behaviors. *Acta Mech. Sin.* **37**, 367-386 (2021).

11 Long, R., Hall, M. S., Wu, M. & Hui, C.-Y. Effects of gel thickness on microscopic indentation measurements of gel modulus. *Biophys. J.* **101**, 643-650 (2011).

12 Carniello, V., Peterson, B. W., van der Mei, H. C. & Busscher, H. J. Physico-chemistry from initial bacterial adhesion to surface-programmed biofilm growth. *Adv. Colloid Interface Sci.* **261**, 1-14 (2018).

13 Cao, P. & Wall, D. The fluidity of the bacterial outer membrane is species specific: bacterial lifestyles and the emergence of a fluid outer membrane. *BioEssays* **42**, 1900246 (2020).

14 Martinac, B. & Kloda, A. Evolutionary origins of mechanosensitive ion channels. *Prog. Biophys. Mol. Biol.* **82**, 11-24 (2003).

15 Zhu, L. *et al.* Gating mechanism of mechanosensitive channel of large conductance: a coupled continuum mechanical-continuum solvation approach. *Biomech. Model Mechanobiol.* **15**, 1557-1576 (2016).

16 Zhu, L., Cui, Q., Xiao, H., Liao, X. & Chen, X. Gating and inactivation of mechanosensitive channels of small conductance: A continuum mechanics study. *J. Mech. Behav. Biomed. Mater.* **90**, 502-514 (2019).

17 Wong, F. & Amir, A. Mechanics and dynamics of bacterial cell lysis. *Biophys. J.* **116**, 2378-2389 (2019).

18 Gibiansky, M. L. *et al.* Bacteria use type IV pili to walk upright and detach from surfaces. *Science* **330**, 197-197 (2010).

19 Conrad, J. C. *et al.* Flagella and pili-mediated near-surface single-cell motility mechanisms in *P. aeruginosa*. *Biophys. J.* **100**, 1608-1616 (2011).
20 Orans, J. *et al.* Crystal structure analysis reveals *Pseudomonas* PilY1 as an essential calcium-dependent regulator of bacterial surface motility. *Proc. Natl. Acad. Sci. U.S.A.* **107**, 1065-1070 (2010).
21 Bohn, Y.-S. T. *et al.* Multiple roles of *Pseudomonas aeruginosa* TBCF10839 PilY1 in motility, transport and infection. *Mol. Microbiol.* **71**, 730-747 (2009).
22 Simsek, A. N. *et al.* Substrate-rigidity dependent migration of an idealized twitching bacterium. *Soft matter* **15**, 6224-6236 (2019).
23 Murray, T. S. & Kazmierczak, B. I. *Pseudomonas aeruginosa* exhibits sliding motility in the absence of type IV pili and flagella. *J. Bacteriol.* **190**, 2700-2708 (2008).
24 Velic, A., Hasan, J., Li, Z. & Yarlagadda, P. K. D. V. Mechanics of Bacterial Interaction and Death on Nanopatterned Surfaces. *Biophysical Journal* **120**, 217-231, doi:10.1016/j.bpj.2020.12.003 (2021).
25 Yao, X., Jericho, M., Pink, D. & Beveridge, T. Thickness and Elasticity of Gram-Negative Murein Sacculi Measured by Atomic Force Microscopy. *Journal of Bacteriology* **181**, 6865-6875, doi:doi:10.1128/jb.181.22.6865-6875.1999 (1999).
26 Gumbart, J. C., Beeby, M., Jensen, G. J. & Roux, B. Escherichia coli Peptidoglycan Structure and Mechanics as Predicted by Atomic-Scale Simulations. *PLOS Computational Biology* **10**, e1003475, doi:10.1371/journal.pcbi.1003475 (2014).
27 Deng, Y., Sun, M. & Shaevitz, J. W. Direct Measurement of Cell Wall Stress Stiffening and Turgor Pressure in Live Bacterial Cells. *Physical Review Letters* **107**, 158101, doi:10.1103/PhysRevLett.107.158101 (2011).
28 Matias, V. R. F., Al-Amoudi, A., Dubochet, J. & Beveridge, T. J. Cryo-Transmission Electron Microscopy of Frozen-Hydrated Sections of <i>Escherichia coli</i> and <i>Pseudomonas aeruginosa</i>. *Journal of Bacteriology* **185**, 6112-6118, doi:doi:10.1128/jb.185.20.6112-6118.2003 (2003).
29 Furchtgott, L., Wingreen, N. S. & Huang, K. C. Mechanisms for maintaining cell shape in rod-shaped Gram-negative bacteria. *Molecular Microbiology* **81**, 340-353, doi:https://doi.org/10.1111/j.1365-2958.2011.07616.x (2011).
30 Kolewe, K. W., Peyton, S. R. & Schiffman, J. D. Fewer Bacteria Adhere to Softer Hydrogels. *ACS Appl Mater Interfaces* **7**, 19562-19569, doi:10.1021/acsami.5b04269 (2015).
31 Nunamaker, E. A., Otto, K. J. & Kipke, D. R. Investigation of the material properties of alginate for the development of hydrogel repair of dura mater. *J. Mech. Behav. Biomed.* **4**, 16-33 (2011).
